# Abomasal dysfunction and cellular and mucin changes during infection of sheep with larval or adult *Teladorsagia circumcincta*

**DOI:** 10.1371/journal.pone.0186752

**Published:** 2017-10-26

**Authors:** Ian Scott, Saleh Umair, Matthew S. Savoian, Heather V. Simpson

**Affiliations:** 1 Institute of Veterinary, Animal and Biomedical Sciences, Massey University, Palmerston North, New Zealand; 2 The Hopkirk Research Institute, AgResearch Ltd, Palmerston North, New Zealand; 3 Institute of Fundamental Sciences, Massey University, Palmerston North, New Zealand; University of Nebraska Medical Center, UNITED STATES

## Abstract

This is the first integrated study of the effects on gastric secretion, inflammation and fundic mucins after infection with L3 *T*. *circumcincta* and in the very early period following transplantation of adult worms. At 3 months-of-age, 20 Coopworth lambs were infected intraruminally with 35,000 L3; infected animals were killed on Days 5, 10, 15, 20 and 30 post-infection and 6 controls on either Day 0 or 30 post-infection. Another 15 Romney cross lambs received 10,000 adult worms at 4–5 months-of-age though surgically-implanted abomasal cannulae and were killed after 6, 12, 24 and 72 hours; uninfected controls were also killed at 72 hours. Blood was collected at regular intervals from all animals for measurement of serum gastrin and pepsinogen and abomasal fluid for pH measurement from cannulated sheep. Tissues collected at necropsy were fixed in Bouin’s fluid for light microscopy, immunocytochemistry and mucin staining and in Karnovsky's fluid for electron microscopy. Nodules around glands containing developing larvae were seen on Day 5 p.i., but generalised effects on secretion occurred only after parasite emergence and within hours after transplantation of adult worms. After L3 infection, there were maximum worm burdens on Days 10–15 post-infection, together with peak tissue eosinophilia, inhibition of gastric acid secretion, hypergastrinaemia, hyperpepsinogenaemia, loss of parietal cells, enlarged gastric pits containing less mucin and increased numbers of mucous neck cells. After adult transplantation, serum pepsinogen was significantly increased after 9 hours and serum gastrin after 18 hours. Parallel changes in host tissues and the numbers of parasites in the abomasal lumen suggest that luminal parasites, but not those in the tissues, are key drivers of the pathophysiology and inflammatory response in animals exposed to parasites for the first time. These results are consistent with initiation of the host response by parasite chemicals diffusing across the surface epithelium, possibly aided by components of ES products which increased permeability. Parietal cells appear to be a key target, resulting in secondary increases in serum gastrin, pit elongation, loss of surface mucins and inhibition of chief cell maturation. Inflammation occurs in parallel, and could either cause the pathology or exacerbate the direct effects of ES products.

## Introduction

Nematodes of the family Trichostrongyloidea which parasitise the abomasum of different ruminants, include *Haemonchus contortus*, *Teladorsagia circumcincta*, *Ostertagia ostertagi*, *Trichostrongylus axei*, *Marshallagia marshalli* and *Ostertagia leptospicularis*. All have both similar life cycles, which differ particularly in the times for emergence from the tissues, and effects on gastric secretion, morphology and mucins, along with a Th2 biased host immune response, which includes tissue infiltration of mast cells and eosinophils [1,2]. Infective third-stage larvae (L3) exsheath in the rumen, then enter abomasal glands where they develop before emerging as L4 or immature adults, causing a raised nodule around the infected gland [3,4]. Generalised effects on secretion, increased abomasal pH and serum gastrin and pepsinogen concentrations, rapidly follow parasite emergence into the abomasal lumen [5–12]. This usually occurs after 5–6 days for *T*. *circumcincta* [5], 2–4 days for *H*. *contortus* [13,14], 5 days for *O*. *leptospicularis* [15], 18 days for *M*. *marshalli* [16] and 16–21 days for *O*. *ostertagi* [6].

Prominent tissue effects are loss of acid-secreting parietal cells and morphological abnormalities in many remaining parietal cells [10,15], although at least some remain viable and capable of responding to stimuli [8,15]. There are also hyperplastic changes, particularly enlarged pits containing less mucin [17,18], and increased numbers of mucous neck cells (MNC) and zymogenic cells with an immature phenotype [11,19]. The control of gastric epithelial cell populations is complex, involving gastrin, the EGF family of peptides and other signalling molecules which maintain the balance between stem cell proliferation in the isthmus and cell death. A pivotal event in the parasitised abomasum is likely to be the inhibition and loss of parietal cells [11,19], which determine the fate of other cell lineages [20–22]. Sheep parietal cells synthesise the transforming growth factor (TGF)-α peptides [23], which include TGF-α, amphiregulin (AR) and heparin-binding epidermal growth factor (HB-EGF) [24–26]. Hypergastrinaemia, resulting from the loss of negative feedback from gastric acidity [8,27–29], stimulates growth of the mucosa and is a potent trophic agent for parietal and ECL cells [30–33], generating new parietal cells in the isthmus. Gastrin increases the expression of HB-EGF and AR [26,34], which promote mucous cell hyperplasia [35,36] and inhibit the differentiation of parietal and zymogenic cells [37]. Mihi et al. [19] have shown increased expression of AR and HB-EGF in bovine abomasal tissues 28 days after *O*. *ostertagi* infection.

The luminal surface of the stomach is covered by a mucus gel formed of alternating layers of Muc5AC, secreted by surface mucus cells (SMC) and pit cells and Muc6 secreted by MNC [38]. In nematode-infected sheep, despite foveolar hyperplasia, expression of Muc5AC is decreased and the mucin content of SMC is markedly reduced, whereas the MNC zone is greatly increased [17,18,39,40]. The significance of the reduced SMC in the parasitised abomasum is unclear, as the opposite occurs in intestinal parasitism. Intestinal mucins play a role in the immunity to nematode parasites through goblet cell hyperplasia and increased secretion of mucus (Muc2) and associated protective proteins, increased mucin sulphation and ectopic expression of gastric type Muc5AC [41]. Critical factors may be the presence or absence of a specific type of mucin or changes in terminal sugars which allow or deter parasite establishment [42]. Thus, reduced secretion of Muc5AC may be permissive for abomasal parasitism and subsequent recovery of its expression may aid in parasite expulsion, or alternatively, a thinner surface mucus gel from reduced Muc5AC expression may be unfavourable for parasite survival and participate in parasite rejection.

Transplantation of adult worms suggests that physical contact with the mucosa or chemicals released by parasites (Excretory/Secretory (ES) products) are responsible at least for initiating the pathophysiology [8–11]. Direct effect of luminal parasites on parietal cell function is supported by the rise in abomasal pH in sheep in which adult worms were confined in porous bags [43], as well as from *in vitro* experiments with tissue preparations. ES products of adult *H*. *contortus* and *O*. *ostertagi* were inhibitory to acid secretion by isolated rabbit gastric glands [44] and cultured parietal cells [19] respectively. Adult *H*. *contortus* ES products also inhibited secretion of histamine (an acid secretogogue) by cultured enterochromaffin-like (ECL) cells [45]. Direct effects of ES products on parietal cells could be enhanced by their ability to increase mucosal permeability [46].

Increased mucosal permeability is a major contributor to hyperpepsinogenaemia associated with parasitism, as well as other forms of gastritis [47,48], and leakage of plasma protein and pepsinogen into the gastric lumen [49,50]. The relative importance of direct effects of ES products, physical contact with parasites and inflammation have not been established. The Th2 response includes infiltration of eosinophils, neutrophils, lymphocytes, mast cells and globule leukocytes and release of cytokines such as interleukin (IL)-1ß and Tumour Necrosis Factor (TNF)-α, which are potent inhibitors of the parietal cell [51,52] and IL-1ß also of the ECL cell [53]. Mihi et al. [19] observed upregulated expression of IL-1ß, IL-8, TNF-α and COX-2 on Day 24 p.i. after a single *O*. *ostertagi* infection and IL-1ß and COX-2 in cultured bovine epithelial cells exposed to adult *O*. *ostertagi* ES products.

The onset of changes in abomasal secretion, epithelial cell populations and mucins and infiltration of eosinophils in sheep infected with either adult or larval *T*. *circumcincta* have been studied in the present experiment to attempt to assess the contributions of parasite and host to the pathophysiology.

## Materials and methods

Animal experiments were carried out in accordance with the requirements of the Massey University Animal Ethics code under the approved protocol MUAEC 97/159, specific for this experiment.

### Animals

26 Coopworth and 15 Romney cross lambs from a Massey University commercial farm were removed from their dams 3 days after birth and reared indoors. They were fed aged lucerne chaff ad libitum and had free access to water. Faecal egg counts were confirmed to be zero before infection. A further 6 sheep were infected with *T*. *circumcincta* L3 to provide adult worms for transplantation into recipient sheep. Animals were killed by exsanguination after stunning with a captive bolt.

Parasite burdens resulting from the dose of L3 were intended to be insufficient to induce overt clinical signs. In our experience such doses are associated with modest declines in feed intake [54], but animals continue to grow and, although faeces may soften, diarrhoea is not seen. In the present study, feed intakes could not be measured since animals were housed in groups, but the study animals gained on average 5 kg by the time of their deaths. The adult transfer experiments were conducted over too short a timeframe for weight loss to manifest, but all animals continued eating and no animals showed any overt clinical signs of parasitism.

### Experimental design

#### Infection with L3

At approximately 3 months-of-age, 20 Coopworth lambs were infected intraruminally with 35,000 *T*. *circumcincta* L3 and 6 remained as uninfected controls. The times of infection were staggered so that one animal was killed per day and animals were killed over a period of 7 weeks. Abomasal tissues were collected at necropsy from infected animals on Day 5 (*N* = 3) and on Days 10, 15, 20 and 30 p.i. (*N* = 4). Control lambs were killed on Day 5 (*N* = 2) and Day 30 p.i. (*N* = 4) and data combined, as data were very similar for all control animals. One animal killed on Day 5 p.i., had significant abomasal pathology unrelated to parasitism, despite appearing to being clinically normal, and was removed from the study.

#### Adult worm transplant

At 4–5 months-of-age, one month prior to transplantation of adult *T*. *circumcincta*, abomasal cannulae were surgically implanted into 15 Romney cross lambs using the procedure previously described by Scott et al. [11]. Ketoprofen (3mg/kg) was administered post-operatively to alleviate pain. The lambs were infected through the cannulae with approximately 10,000 adult *T*. *circumcincta* and killed after 6, 12, 24 and 72 h (*N* = 3). Uninfected controls (*N* = 3) were also killed at 72 h.

### Parasitology

Infective L3 were cultured from faeces from sheep infected with a pure strain of *T*. *circumcincta* and stored at 4 ^o^C prior to use. Faecal egg counts per g faeces were determined using a modified McMaster method [55]. Luminal worm counts were carried out on 10% of the abomasal contents.

Adult worms were obtained from 6 donor sheep infected with approximately 50,000 *T*. *circumcincta* L3 and killed on Day 21 p.i. The abomasa were opened, washed with PBS and pooled contents and washings were allowed to sediment. After discarding the superficial fluid, the parasite suspension was divided into 13 aliquots: 12 for infecting recipient animals and one for a worm count.

### Blood and abomasal fluid samples

Abomasal fluid samples were collected only at necropsy from animals infected with L3. Samples (20 ml) were collected via the cannulae from animals receiving adult worms 1 and 4 days and immediately prior to infection, and 6, 9, 12, 15, 18, 21, 24, 30, 36, 42, 46, 49, 53, 62 and 72 h p.i (S2 Table). The pH was measured using a PHM82 Standard pH Meter (Radiometer, Copenhagen, Denmark).

Jugular blood was collected from animals infected with L3 before the start of the experiment, daily until Day 15 p.i., then approximately every second day until Day 30 p.i. (S1 Table). Blood samples were taken after adult transplant at the times when abomasal fluid was collected. Blood was collected into plain evacuated tubes, allowed to clot and then spun at 2000 *g* to collect serum, which was stored in aliquots at -20°C.

### Necropsy and tissue collection

Animals were killed by exsanguination following stunning with a captive bolt. The abomasum was removed, the contents were collected and the abomasum was opened along the greater curvature. Tissue (1cm^2^) was collected for light microscopy from each of 5 of the fundic spiral fundic folds and from pyloric mucosa 5–10 cm from the sphincter and smaller pieces were fixed in Karnovsky's fluid for electron microscopy. The mucosal surface was then washed with 500 ml of warmed Ringer’s solution (122 mM NaCl, 25 mM NaHCO_3_, 5 mM KCl, 1.3 mM MgSO_4_, 2.0 mM CaCl_2_, 1.0 mM KH_2_PO_4_ and 20 mM glucose) and the washings added to the contents for performing worm counts.

### Serum gastrin and pepsinogen assays

Serum gastrin concentrations were determined in triplicate by a radioimmunoassay [56] based on the method of Hansky and Cain [57]. Synthetic human nsG17 (Research Plus, USA) was used to prepare radiolabel and standards. Pepsinogen concentrations were determined by a method validated by Scott et al. [58], with minor modifications [59]. Peptic activity was calculated from the tyrosine-containing peptide fragments liberated by digestion of glycine-buffered BSA, following the conversion of pepsinogen to pepsin at pH< 2.

### Histology

Tissue fixed in Bouin's fluid was routinely dehydrated and embedded in paraffin using an automatic tissue processor (SE400, Shandon Scientific Co., UK). Sections were cut at 5 μm thick and stained with haematoxylin and eosin (H & E) for histopathological examination, with toluidine blue for mast cells, immunohistochemically for eosinophils and parietal cells and also with mucin stains. Tissues fixed in Karnovsky's fluid were routinely processed and embedded in epoxy resin (Epon 812). Sections for transmission electron microscopy were cut 70–90 nm thick, mounted on 200 mesh copper grids (Alltech, NZ) and stained with 2% uranyl acetate followed by Reynold’s lead citrate [60].

### Immunohistochemistry

Parietal cells were labelled immunohistochemically for both TGF-α and the proton pump (H^+^/K^+^-ATPase). For TGF-a staining, a mouse monoclonal antibody (IgG_2a_) produced against recombinant human TGF-α (Calbiochem, USA) was used at a 1:500 dilution in PBS containing 0.5% BSA. The pump antibody, a mouse monoclonal (IgG1) against the β-subunit of porcine H^+^/K^+^-ATPase (Pierce Biotechnology, USA), was used at a 1:4000 dilution. Eosinophils were visualised with a mouse monoclonal (IgG_1_) antibody (the kind gift of Dr. Wayne Hein, AgResearch Ltd, Wallaceville, NZ) at 1:1000 dilution. All 3 primary antibodies were used with an anti-mouse IgG Vectastain Elite ABC kit (Vector Laboratories, USA) and diaminobenzidine as the chromogen. All sections were counterstained with Mayer’s haematoxylin.

### Mucin histology

Fundic tissues were stained with Periodic Acid Schiff (PAS) for all mucins, Alcian Blue (AB)/PAS pH 2.5 for both sialylated and suphated mucin and High Iron Diamine (HID) and AB/PAS pH 1 for sulphated mucin [61]. Inclusion of AB/PAS pH 1 in the protocol allows distinction of sialomucins and sulphomucins. Lectin histochemistry was performed with UEA-1 (*Ulex europaeus* agglutinin-1) for α-1,2-linked Fuc [18]. Sections were imaged under bright field illumination, using an Olympus BX51 microscope outfitted with a MicroPublisher 5 colour camera (Q-Imaging) and running QCapture PRO 7 software. Captured images were flatfield corrected and white balanced prior to figure generation in Photoshop CC (Adobe Systems Inc.).

### Cell counts

Eosinophils, nucleated TGF-α-labelled parietal cells and mast cells in areas where the fundic glands were orientated in longitudinal section were counted at ×400 magnification, using a 1 cm^2^ eyepiece graticule. Counts were made at 5 locations in each section in a 258 μm wide column of mucosa from the muscularis mucosae to the luminal surface, avoiding areas with obvious nodules. Mucosal thickness was also measured directly at 2 locations in each of 2 separate sections of fundic tissue. Counts of eosinophils and mast cells in pyloric tissues were made at up to 5 locations in one section from each animal.

### Data analysis

Data were plotted using Graphpad Prism v5. Eosinophil (counts + 1) and mast cell numbers were subjected to Log_10_-transformation prior to analysis. Abomasal pH for the adult transplant experiment was analysed by two-way ANOVA and comparison of means at each time point with pre-infection and between infected and control animals at each time point. All other group data were analysed by one-way ANOVA, followed by Dunnett’s tests to compare means at each time point with uninfected controls, using either Minitab release 16 (Minitab Inc., USA) or Graphpad Prism.

## Results

### Infection with *T*. *circumcincta* L3

#### Abomasal secretion

**Fig 1:**
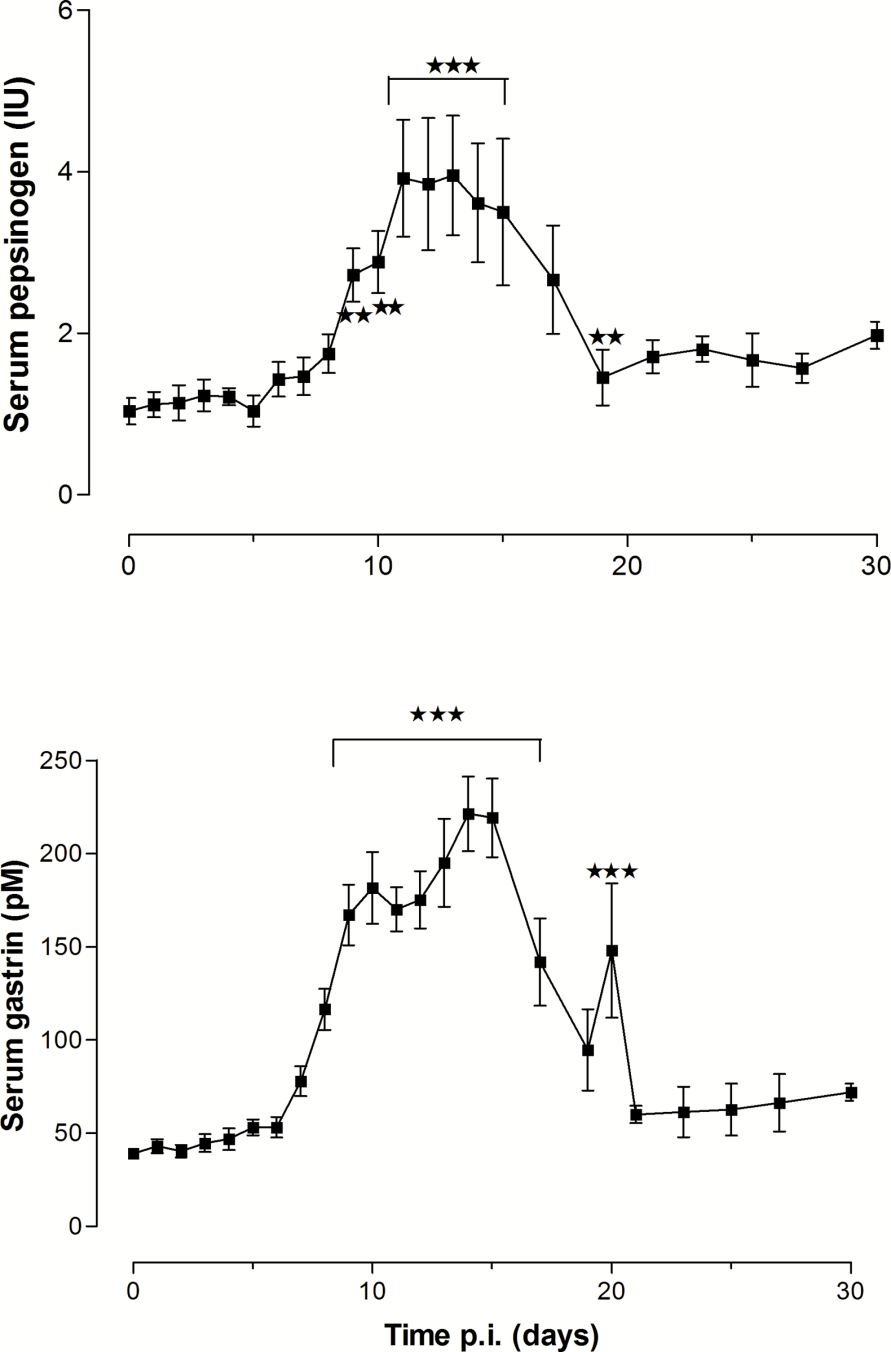
Serum concentrations of pepsinogen (IU) (top) and gastrin (pM) (bottom) (mean ± SEM) in lambs infected with 35,000 *Teladorsagia circumcincta* L3. The maximum number of replicates was *N* = 20, reducing by 4 at Days 5, 10, 15 and 20 p.i. as animals were euthanased.

The pH of abomasal contents measured at necropsy was significantly increased on Days 10 and 15 p.i. (p<0.01) and returning to pre-infection levels on Days 20 and 30 p.i. (Table 1). Serum gastrin and pepsinogen levels, monitored over the whole period (Fig 1) were significantly raised (p<0.01 or p<0.001) between Days 8 and 20 p.i. and Days 9 and 20 p.i. respectively. Data for one animal were omitted from the group data, as this animal had an atypical late rise in serum gastrin from Day 20 p.i. to 740 pM 3 days prior to necropsy on Day 30 p.i.

**Table 1:** Abomasal fluid pH, fundic mucosal thickness (μm), parietal cells, eosinophils and mast cells per 258 μm wide column in the fundus and pylorus of uninfected control animals and lambs euthanased on Days 5, 10, 15, 20 and 30 after infection with 35,000 L3 *T*. *circumcincta*.

Time p.i. (days)	Abomasal pH	Mucosal thickness (μm)	Parietal cells per	Eosinophils per column		Mast cells per column	
	mean ± SD (*N*)		column	mean (CI; *N*)		mean (CI; *N*)	
			mean ± SD (*N*)				
		mean ± SD (*N*)		Fundus	Pylorus	Fundus	Pylorus
Control	2.7 ± 0.2 (5)	435.9 ± 22.5 (6)	141.5 ± 9.8 (6)	0.8	14.7	7.8	14.5
				(0.09–1.94; 6)	(4.1–53.0; 6)	(6.6–9.3; 6)	(10.5–20.1; 6)
5	2.8 ± 0.3 (3)	415.4 ± 126.7 (3)	145.1 ± 25.4 (3)	0.8	6.6	7.7	10.9
				(-0.33–3.86; 3)	(1.0–42.9; 3)	(6.0–10.0; 3)	(5.4–22.2; 3)
10	5.3 ± 0.7** (4)	616.9 ± 45.7** (4)	109.9 ± 14.0* (4)	32.8**	20.7	11.4	17.7
				(20.7–51.5; 4)	(0.4–95.2; 4)	(6.2–20.8; 4)	(12.6–24.7; 4)
15	4.5 ± 0.3** (4)	569.7 ± 56.0* (4)	83.8 ± 11.9** (4)	16.9**	36.7	14.7	20.8
				(8.7–32.0; 4)	(7.0–192.7; 4)	(5.1–42.1; 4)	(13.9–31.3; 4)
20	3.6 ± 0.3 (4)	538.5 ± 23.4 (4)	121.4 ± 23.0 (4)	11.0**	36.5	15.2	23.5
				(2.6–40.3; 4)	(14.0–95.1; 4)	(5.5–42.2; 4)	(16.7–33.0; 4)
30	3.4 ± 0.2 (4)	572.4 ± 130.8* (4)	150.2 ± 13.6 (3)	5.7**	15.4	25.1**	23.4
				(2.1–13.3; 4)	(0.1–293.1; 4)	(11.0–57.4; 4)	(4.3–126.2; 4)

Data are presented as mean ± SD (*N*) or as geometric mean, 95% confidence interval and n for eosinophils and mast cells.

Significantly different means from control are shown

*p<0.05.

**p<0.01.

#### Parasitology and gross pathology

Worm counts of 15,860 and 10,200 on Day 10 p.i. were >90% immature adults and 10% late L4. Adult worms were present on Days 15–30 p.i.: 20,960, 15,620 (Day 15 p.i.), 1,600, 13,280, 9,800, 2,500 (Day 20 p.i.) and 400, 400, 200 and 700 (Day 30 p.i.).

The mucosal surface was smooth with few irregularities in control animals and the 2–3 cm deep spiral folds had little fatty sub-mucosal tissue between the two mucosal layers. Nodules were present on Day 5 p.i., predominantly in the pylorus, with fundic nodules (S1 Fig) mainly at the aboral ends of the folds. On Days 10–20 p.i., there was considerable sub-mucosal oedema, visible as a gel-like material underlying the mucosa in the fundic folds, visible nodules mostly in the pylorus and much of the fundic mucosa showed generalised thickening with a roughened and pale surface. By Day 30 p.i., tissues appeared more normal, with visible thickening only in one animal.

Histological measurement of the fundic mucosal depth, showed it was significantly increased from Days 10 to 30 p.i. (Table 1).

#### Parietal cells

**Fig 2:**
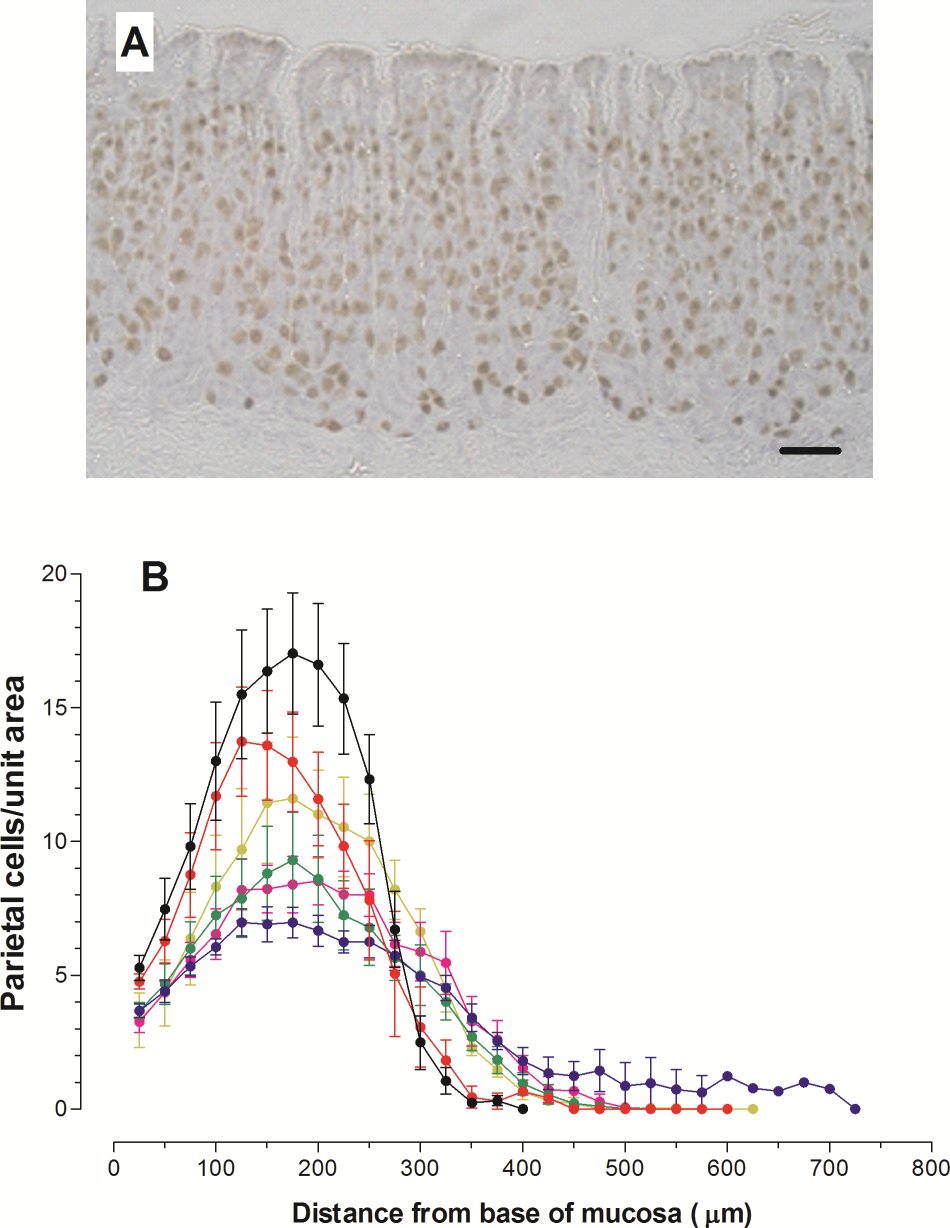
Parietal cell profiles in the fundus of infected animals. (A) section of fundic mucosa of an uninfected animal showing immunohistochemical staining of parietal cells with anti-TGF-α antibody. Bar = 50 μm. (B) Profiles of parietal cells in 258 μm wide columns of tissue in uninfected animals and lambs euthanased on Days 5, 10, 15, 20 and 30 after infection with 35,000 L3 *Teladorsagia circumcincta*. Data are expressed as mean ± SD. Symbols: •: uninfected; red o: Day 5; blue o: Day 10; green o: Day 15; pink o: Day 20; yellow o: Day 30 p.i.

As parietal cell counts of cells stained with either anti-TGF-α (Fig 2A) or anti-pump antibodies (S2 Fig) were similar, only data from TGF-α-positive parietal cells are presented. Parietal cells were located from the progenitor zone down to the base of the mucosa and occasional cells migrated upward into the pits. The profiles of parietal cell densities in Fig 2B shows the enlarged pit zone and elongated glands in infected animals and depletion of parietal cells at all levels, particularly in the mid-gland region. The total number of parietal cells per 258 μm wide column of mucosa was significantly reduced on Days 10 (p<0.05) and 15 p.i. (p<0.01) (Table 1).

**Fig 3:**
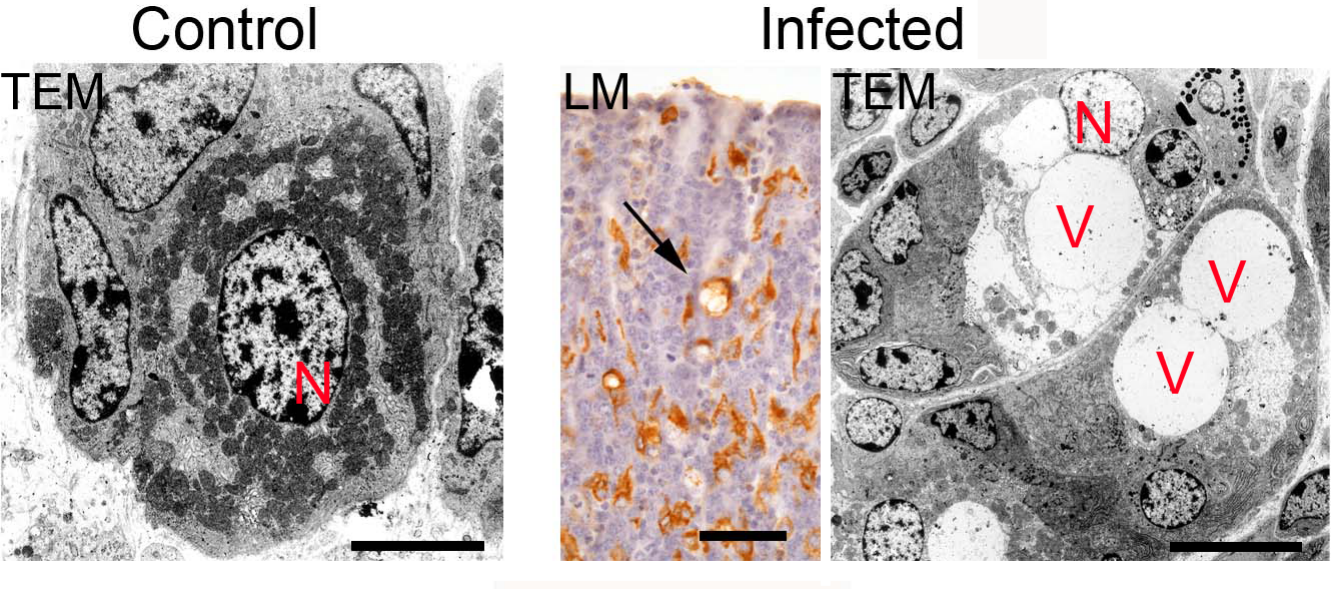
Parietal cells from an uninfected control animal (left) and from a lamb euthanased on Day 10 p.i. after infection with 35,000 L3 *Teladorsagia circumcincta* (centre and right). Left: TEM showing a central nucleus (N), cytoplasm filled with mitochondria and visible intracellular canaliculi; centre: LM section showing parietal cells stained brown with anti-H^+^/K^+^-ATPase antibody and a vacuolated parietal cell (arrow); right: TEM of parietal cells containing one or more vacuole-like structures (V), pale cytoplasm, reduced numbers of mitochondria and swollen pale nuclei. Bar = 5 μm (left, right). 50 μm (centre).

There was focal depletion of parietal cells within nodules in the fundus on Day 5 p.i. and on Day 10 p.i. there were areas of parietal cell loss which appeared unrelated to the localised hyperplasia of a nodule (S2 Fig). By Day 10 p.i., many parietal cells were abnormal; some were elongated and many contained vacuole-like structures (S2 Fig). These abnormal cells stained with both anti-TGF-α and proton pump antibodies. Ultrastructurally abnormal parietal cells had swollen pale nuclei, pale cytoplasm and reduced numbers of mitochondria and were seen on Day 10 p.i. and thereafter (Fig 3).

#### Inflammatory cells

The very small numbers of eosinophils in uninfected fundic tissues were generally located at the mucosal base, adjacent to the muscularis. There were more eosinophils in the pylorus, consistent with higher cell counts both before and after infection (Table 1). On Day 5 p.i., the small number of larvae detected within the fundic sections examined were associated with focal accumulations of eosinophils, neutrophils and small numbers of lymphocytes within typical hyperplastic nodules. Although nodules were infiltrated by increased numbers of eosinophils, most remained in the base of the mucosa distant from the parasitised gland at the heart of the nodule.

**Fig 4:**
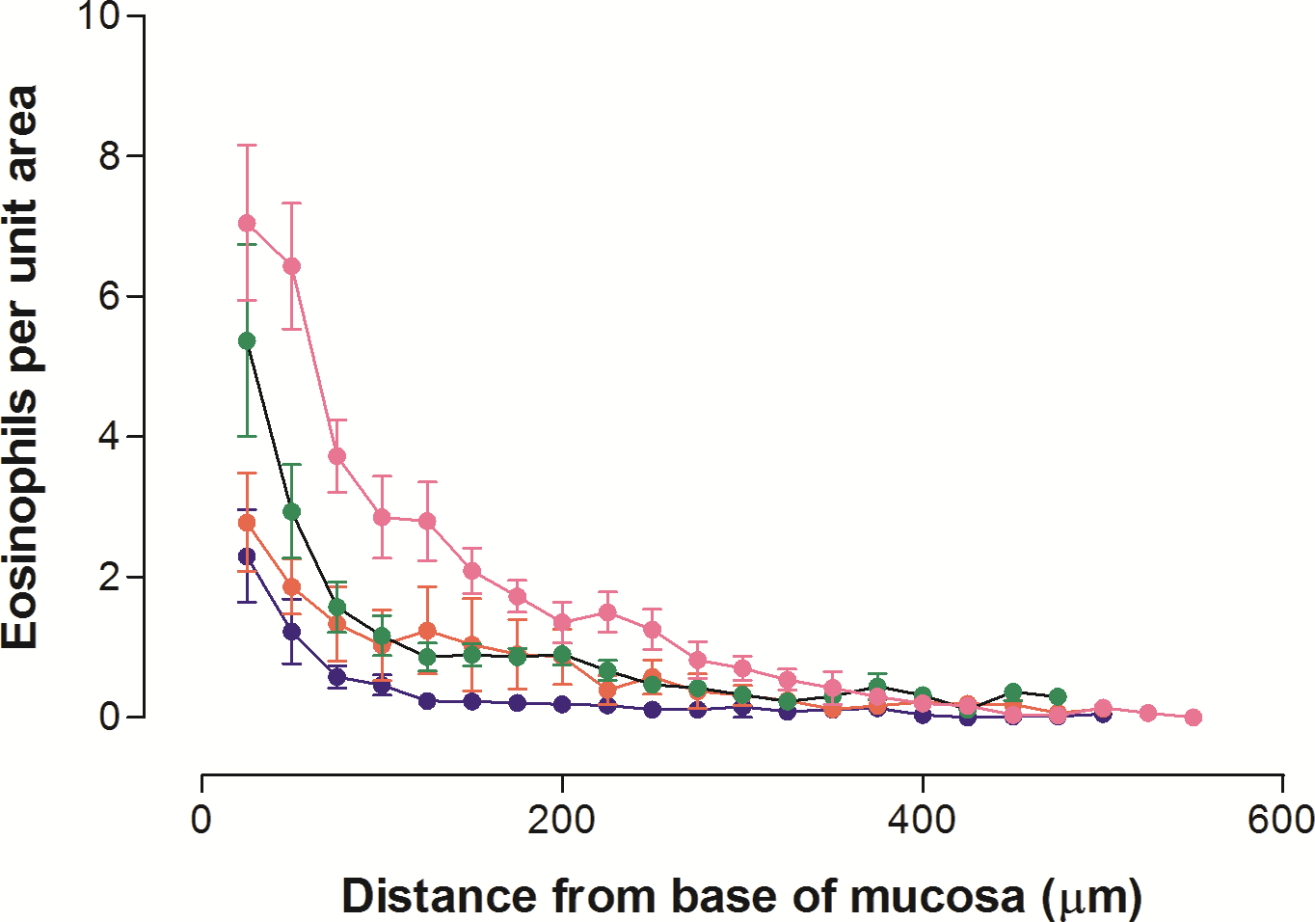
Profiles of eosinophils in 258 μm wide columns of fundic tissue from lambs euthanased on Days 10, 15, 20 and 30 after infection with 35,000 L3 *Teladorsagia circumcincta*. Data are expressed as mean ± SD. Symbols: pink o: Day 10; green o: Day 15; red o: Day 20; blue o: Day 30 p.i.

From Day 10 p.i., there was a marked, generalised influx of eosinophils into the lamina propria of the mucosa and into the sub-mucosa. The profile of tissue eosinophils in the fundic mucosa showed higher numbers in the base of the mucosa and few in the mid- and upper glands and pits, as well as the peak numbers on Day 10 p.i., decreasing to Day 30 p.i. (Fig 4). This is consistent with the total counts of the eosinophils per column of mucosa (Table 1), which were greater than control from Day 10 p.i. (p<0.01), when there was the highest mean, followed by a subsequent decline to Day 30 p.i.

Mast cells were scattered evenly throughout both the fundus and pylorus. Fundic mast cell numbers were significantly greater than in uninfected animals on Day 30 p.i. (Table 1). Globule leukocytes were not enumerated, but appeared to be prominent only on Day 30 p.i. in 3 of the 4 animals, of which two had scattered clusters of small numbers of cells, whilst the third had cells present within the epithelium of many of the pits.

#### Fundic mucins

**Fig 5:**
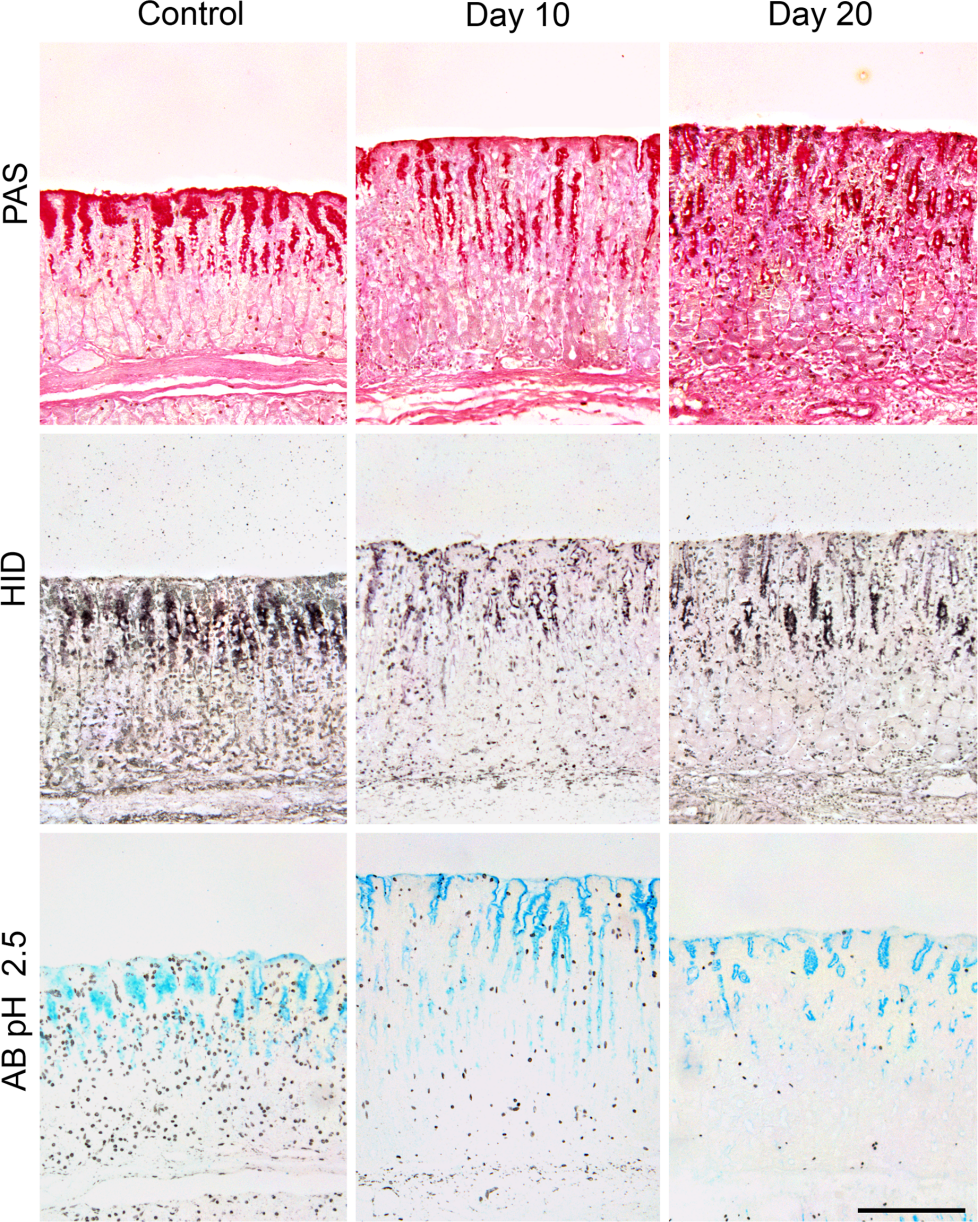
Fundic tissues of uninfected control sheep (left panel) and on Day10 p.i. (centre panel) or Day 20 p.i. (right panel) after infection with 35,000 L3 *Teladorsagia circumcincta*. Stains used: (top) Periodic Acid Schiff for all mucins; (centre) High Iron Diamine for sulphated mucins (bottom) Alcian Blue pH 2.5 for both sialylated and sulphated mucin. Scale bar: 200 μm.

In uninfected animals, there was strong staining of mucins in SMC, on the luminal surface and in cells in the short, wide pits. The pits were filled with PAS-positive material and clearly delineated from the small zone of scattered MNC, which also contained mucins (Fig 5). Sulphated mucins, stained either with HID or AB pH 1, were present in the mid- to lower pits and MNC, but not in the SMC. Sialomucins stained more strongly in the pits and glands, with less in SMC and on the luminal surface.

Infection caused no marked changes in mucins on Day 5 p.i. (Fig 5), although there were variations in intensity of staining between animals. By Day 10 p.i., mucins were almost absent from the SMC and the luminal surface (Fig 5 and S3–S7 Figs) and there was less intense staining of pit cells, especially the upper regions. The MNC population was expanded to about 50% depth of the glands and stained moderately with PAS. These changes persisted until Day 30 p.i. in most animals, but there was evidence of recovery of mucin production in the pits and SMC, particularly in one sheep which had largely recovered. Staining with AB pH 2.5 for acidic (sialylated and sulphated) mucins varied considerably in individual animals, generally becoming paler from Day 10 p.i onward. MNC were moderately stained, whereas SMC staining became less. HID staining showed reduced sulphation of mucins in pit cells from Days 10–15 p.i. and a tendency for increases again on Days 20 and 30 p.i.

### Transplantation of adult *T*. *circumcincta*

#### Abomasal secretion

**Fig 6:**
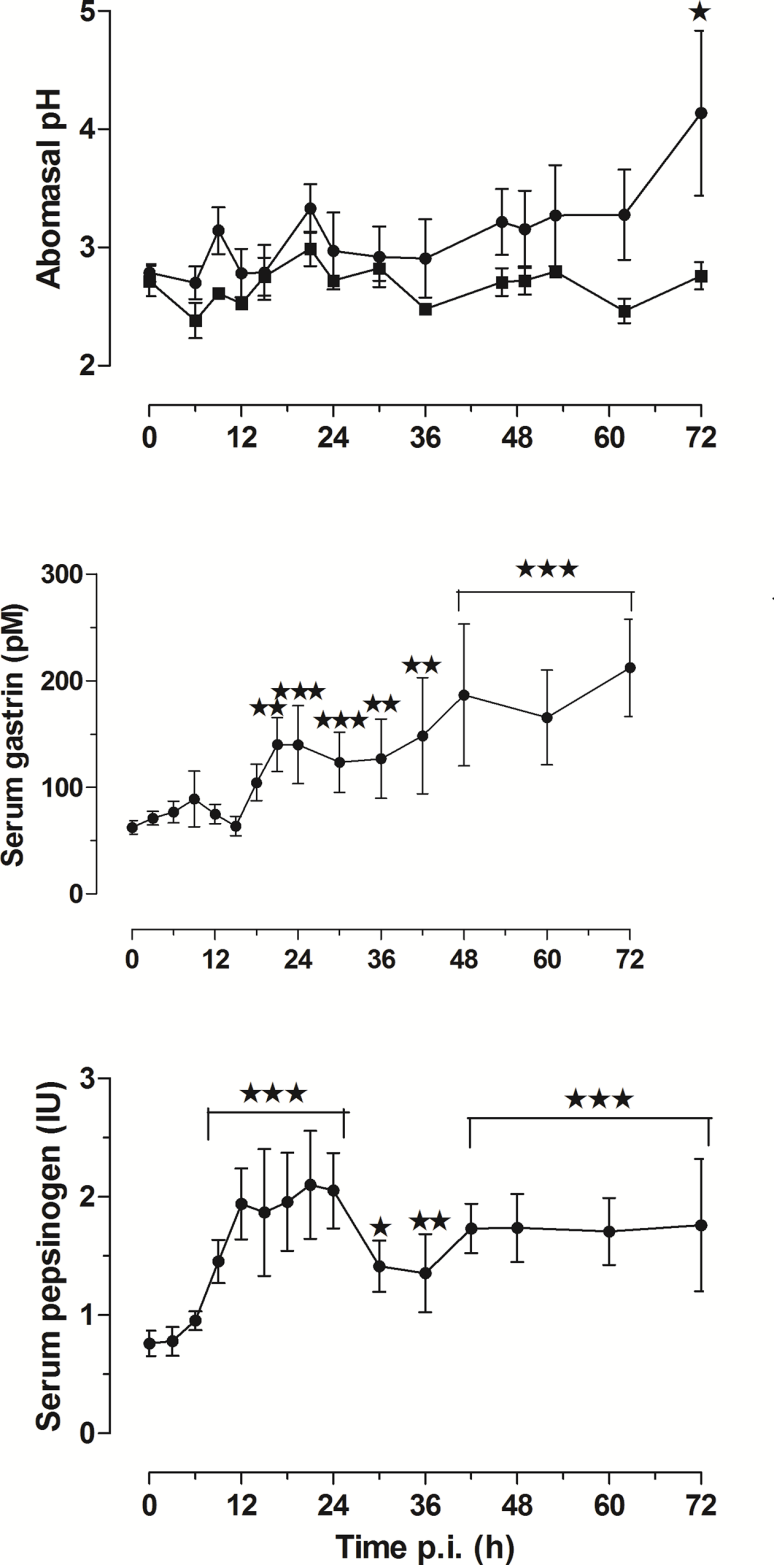
pH of abomasal contents and serum gastrin and pepsinogen concentrations (pM) (bottom) (mean ± SEM) in lambs transplanted with 10,000 adult *Teladorsagia circumcincta*. The maximum number of replicates was *N* = 12, reducing by 3 at 6, 12, and 24 h as animals were euthanased. Significant differences from time zero are shown: *p<0.05; **p<0.01; ***p<0.001.

The pH of abomasal contents and serum and pepsinogen concentrations of infected sheep were monitored frequently over the 72 h (Fig 6). The number of replicates decreased from an initial *N* = 12 as animals were euthanased in groups of 3. All parameters were significantly increased by the parasites. The mean abomasal pH of infected and controls slowly diverged and overall the pH was raised in infected lambs (p<0.001) and at 72 h (p<0.05). Serum pepsinogen concentration began increasing from 6 h p.i., reached a plateau at 15 h and was significantly increased from 9 h p.i. Serum gastrin concentrations were significantly increased from 18 h p.i. and continued to increase slowly to the end of the experiment.

#### Parasitology and gross pathology

Worms were not enumerated, but were visible in all infected animals closely adhering to the mucosal surface in both the fundus and pylorus. Parasites were not evenly dispersed and appeared to be associated with irregular areas of mild hyperplasia and sub-mucosal oedema in some, but not all, infected animals.

#### Histopathology

Areas of parietal cell loss were apparent in sections of the fundus in animals killed 72 h after transplant of adult worms (S2 Fig). No globular leukocytes were seen, but eosinophils, lymphocytes and neutrophils were present. None of the total cell counts per 258 μm column of mucosa or mucosal thickness were statistically significantly different from control (Table 2) and varied between animals, although there appeared to be a trend for increases in fundic thickness and eosinophil numbers and a decrease in parietal cell numbers at 72 h (*N* = 3). Mast cell numbers appeared unaffected by the transplantation of adult parasites.

**Table 2:** Fundic mucosal thickness (μm) and numbers of parietal cells, eosinophils and mast cells per 258 μm wide column (all *N* = 3) in uninfected animals and at 6, 12, 24 and 72 hours after infection with 10,000 adult *Teladorsagia circumcincta*.

Time p.i.	Mucosal	Parietal cells	Eosinophils	Mast cells
(h)	thickness (μm)	per column	per column	per column
Uninfected	384.4±24.0	129.7±6.8	0.2(0.1–0.2)	3.7(1.2–11.4)
6 h	383.6±36.8	159.3±1.8	3.0(-0.8–76.4)	2.6(1.0–6.5)
12 h	409.1±32.1	149.7±30.1	3.7(-0.6–49.5)	3.2(2.1–4.8)
24 h	385.5±35.3	164.7±20.3	2.5(-0.4–19.7)	5.9(2.7–12.8)
72 h	464.8±38.3	111.3±11.8	10.6(-0.5–294.4)	2.6(1.3–5.5)

Data are presented as mean ± SD, except for eosinophil and mast cells (geometric mean and 95% confidence interval).

#### Fundic mucins

**Fig 7:**
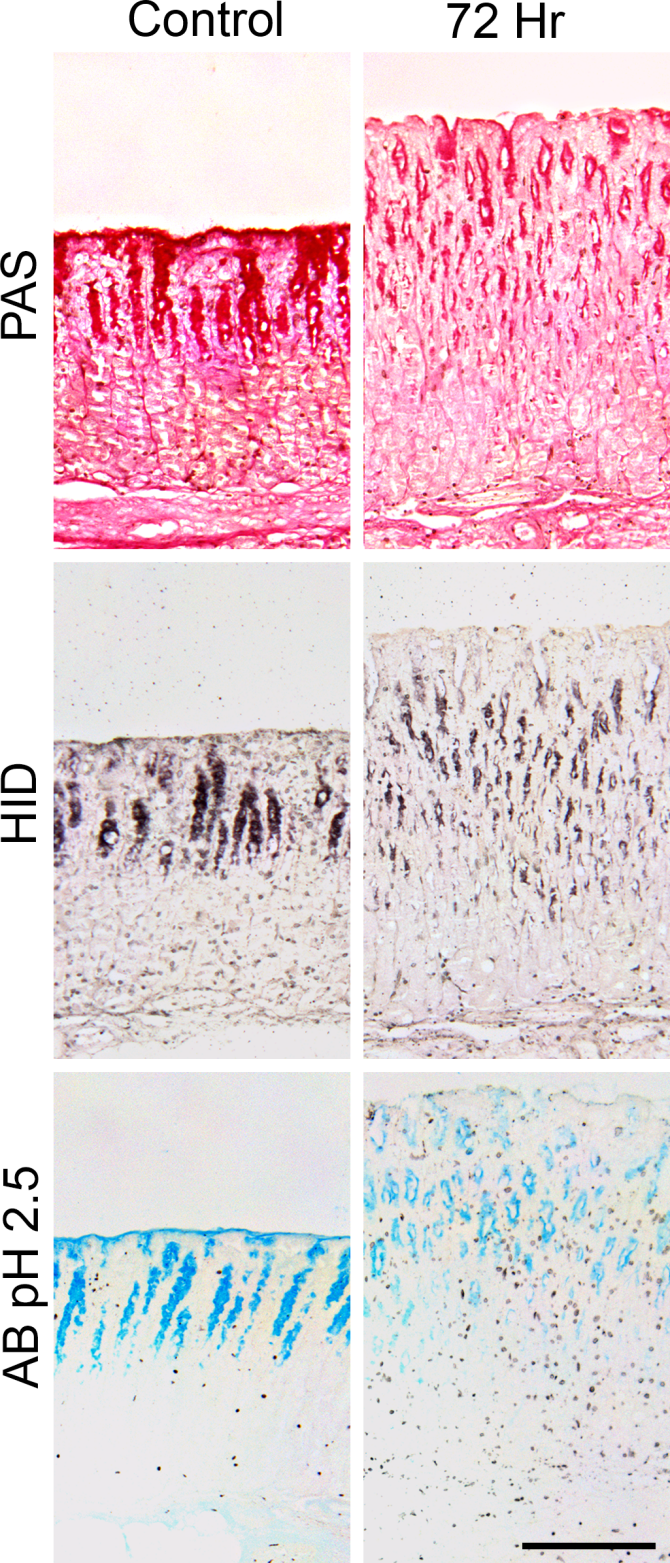
Fundic tissues of uninfected sheep (left panel) and 72 hours after transplantation of 10,000 adult *Teladorsagia circumcincta* (right panel). Stains used: (top) Periodic Acid Schiff for all mucins; (centre) High Iron Diamine for sulphated mucins and (bottom) Alcian Blue pH 2.5 for both sialylated and sulphated mucin. Scale bar: 200 μm.

Visible changes were apparent in tissues by 24 h p.i., particularly reduced surface mucus and expansion of the MNC zone. HID staining for sulphomucins was clearly reduced at 24 h p.i. in one sheep. By 72 h after adult transplant, tissues from all 3 sheep resembled those after L3 infection, with marked loss of pit cell sulphated mucins, in parallel with reduced staining with PAS (Fig 7).

Raw data are presented in S1 Table and S2 Table.

## Discussion

This is the first integrated study of the effects on gastric secretion, inflammation and fundic mucins during one lifecycle of *T*. *circumcincta* over 30 days after L3 infection and also in the very early period following transplantation of adult worms through an indwelling abomasal cannula. The striking finding was that all parameters changed in parallel to the numbers of parasites in the abomasal lumen, suggesting that these lifecycle stages are key drivers of the pathophysiology and inflammatory response.

### Inflammation

Parasites remained in the tissues up to Day 5 p.i., when nodules around developing larvae were visible, predominantly in the pylorus, whilst the few nodules present in the fundus tended to be on the aboral ends of fundic folds. This pattern may be somewhat atypical, but was also seen in the animals studied by Somerville [3]. Inflammatory cells accumulated at the periphery of nodules, but not in contact with larvae, but at all times eosinophils remained in the basal mucosa closest to the muscularis (Fig 4), suggesting that, at least in previously parasite-naïve animals, eosinophils recruited into tissues do not directly attack the parasites. Balic et al. [62], also noted lack of contact of inflammatory cells with larvae in naïve *H*. *contortus*-infected sheep, whereas, inflammatory cells were able to surround and penetrate glands harbouring larvae in immune animals.

The major influx of eosinophils into the fundus of larval-infected animals between Days 5 and 10 p.i. (Table 1) coincided with the presence in both the fundus and pylorus of luminal worms (>90% immature adults and 10% late L4). *T*. *circumcincta* typically emerge from the mucosa around Days 5–6 p.i. [5,8]. Emerged worms were seen from Day 10 p.i. onward, closely adhering to the mucosal surface in both the fundus and pylorus. As expected in unselected animals, there were variable worm burdens up to 20,000, as genetics plays an important part in determining both resistance to parasitism [63] and resilience to their effects [54]. Parasites were already being lost by Day 20 p.i. and by Day 30 p.i., less than 5% of the population remained. Tissue eosinophil numbers declined in parallel, but mast cell numbers remained elevated (Table 1), and gross pathological changes had resolved in all but one animal by Day 30 p.i. Following the transplantation of adult parasites, the influx of eosinophils was seen as early as 6 hours p.i. in some sheep, but was quantitatively very variable (Table 2). Eosinophils may be attracted by the release of eosinophil chemotactic factors by *T*. *circumcincta* [64,65], as also occurs with other nematodes, including *O*. *ostertagi* [66] and *H*. *contortus* [64,67], or by chemokines originating from host epithelial or inflammatory cells in response to physical or chemical stimuli from the parasites [68–70].

### Abomasal secretion

The presence of parasites in the lumen, either immature adults after L3 infection or transplanted mature adult worms, increased abomasal pH and serum gastrin and pepsinogen concentrations. Serum pepsinogen was significantly increased within 9 hours and gastrin in 18 hours after adult transplantation (Fig 6) and at the predicted time of emergence of larvae around Days 5–6 p.i., as has been previously reported [5–11]. The rapid effect of luminal parasites could be seen as evidence that parasite chemicals entering the tissues through the the surface epithelium of the abomasum initiate the effects on secretory cells, probably augmented by associated inflammation. Release of chemicals as a result of physical or chemical effects of worms on the surface epithelium could also play a part, as cultured epithelial cells can both release endogenous cytokines when exposed to bacterial pathogens [71] and are responsive to exogenous cytokines [72]. The entry of secreted chemicals may be facilitated by worm secretions increasing epithelial permeability; ES products of both *T*. *circumcincta* and *H*. *contortus* increased epithelial permeability of model epithelial cells (Caco-2 cells) *in vitro* [46].

The relative contributions of inflammation and direct effects of ES products remain unclear, as effects on abomasal secretion changed in parallel after L3 infection both with luminal worm numbers and inflammation, as assessed by fundic tissue eosinophilia. Parasite ES products may directly affect the functioning of abomasal cells, particularly parietal cells and ECL cells, which ES products can inhibit *in vitro* [19,44,45]. In contrast, ES products of *H*. *contortus* [73] or *T*. *circumcincta* [74] were ineffective in stimulating gastrin secretion by the G-cell.

Inflammation may be pivotally involved in parietal cell inhibition, as abomasal pH did not rise in sheep in which fundic eosinophil numbers remained low and a rise in pH was not seen in the absence of inflammation. As in *O*. *ostertagi* infection [19], the infiltration of eosinophils and other inflammatory cells would release cytokines, including the potent inhibitors of parietal and ECL cells, IL1-ß and TNF-α [51–53]. Hypergastrinaemia probably results largely from loss of negative feedback from gastric acidity [8,27–29], but may be exacerbated by inflammation, as gastrin secretion is stimulated by inflammatory mediators, including histamine [75], TNF-α [76,77] and IL-1β [77]. A delayed and exaggerated loss of acid feedback is likely the cause of the unusual late peak in serum gastrin in the animal whose gastrin data were excluded from the analysis, as it also had the lowest parietal cell counts and thickest and heaviest mucosal tissues, both indicators of hypergastrinaemia.

Increased circulating pepsinogen levels and increased leakage of plasma protein into the gastric lumen are typical of parasitised animals [49,50] and are probably caused more by inflammation than by worm chemicals. Hyperpepsinogenaemia is considered to be evidence of a leaky abomasal mucosa in many forms of gastritis and is used as a marker for human *Helicobacter pylori* infection [47,48]. Eosinophils accumulated at the base of the gastric glands (Fig 4) where the chief cells are located, supporting a role for greater permeability in the gland region where pepsinogen is secreted. Whilst raised circulating levels are thus largely a result of inflammation, reduced conversion of pepsinogen to pepsin at raised abomasal pH [78] and increased secretion of pepsinogen may be contributing factors [79,80], as inflammatory cytokines and leukotrienes [81] and elevated gastrin levels [82] stimulate chief cells.

### Fundic tissue architecture

Inhibition and loss of parietal cells, which determine the fate of other cell lineages [20–22], are probable causes of increased tissue thickness and changes in mucus-secreting cells apparent by Day 10 p.i. The hypergastrinaemia would aid recovery from parietal cell loss by stimulating mucosal growth and generating new parietal cells in the isthmus [30–33] and increasing the expression of HB-EGF and AR [26,34], which promote mucous cell hyperplasia [35,36] and inhibit the differentiation of parietal and zymogenic cells [37]. Increased expression of AR and HB-EGF occurs in the bovine abomasum 7 days after emergence of *O*. *ostertagi* [19].

There was significant loss of parietal cells (22% and 41% on Days 10 and 15 p.i. respectively) after larval infection (Table 1 and Fig 2), as identified by staining for TGF-α [23]. The proton pump remained antigenic in abnormal cells, and a similar number of parietal cells were counted using antibodies to either H^+^/K^+^-ATPase or TGF-α. Parietal cell death by necrosis has previously been reported for *T*. *circumcincta* infection of sheep [11] and calves infected with *O*. *ostertagi* [83] or *T*. *axei* [84] and accompanies disruption of the Na^+^/H^+^ exchanger in transgenic mice [85] and in rabbit gastric glands after acid inhibition with omeprazole [32] or ammonia [86].

Many remaining parietal cells were morphologically abnormal, with swollen pale nuclei, pale cytoplasm, fewer mitochondria and many also with one or more large vacuole-like structures (Fig 3). Dilation of the intracellular canaliculi was not a feature in the present study, although seen in an earlier study of tissues collected by biopsy [11]. Dilated canaliculi are an early effect of pharmacological inhibition by ranitidine [87], omeprazole [32,88] and atropine [89], which may progress to condensation of canaliculi to form large vacuole-like structures associated with further degeneration of the parietal cells [87]. It is possible that parasite ES products may participate in the vacuolation process, as *T*. *circumcincta* and *H*. *contortus* chemicals promote vacuolation of HeLa cells *in vitro* [90,91]. Vacuolation is also a feature of infections of gastric tissue by *H*. *pylori* via a secreted cytotoxin (VacA), although the mechanisms of HeLa cell vacuolation due to VacA and nematode ES products appear to differ [91].

### Gastric mucin

After either larval or adult worm infection, there were reduced total mucin and sulphomucins in SMC and pit cells and an expanded the MNC zone as seen in earlier studies at a small number of time points in an infection [18,92,93]. These changes in gastric mucins (Figs 5 and 7 and S3–S7 Figs) also followed the same time course as effects on abomasal secretion, tissue eosinophilia and the numbers of luminal worms, becoming marked from Day 10 p.i. In uninfected animals, there was strong staining of Muc5AC in SMC, on the luminal surface and in cells in the short, wide pits, but few MNC which synthesise Muc6 (Fig 5) [18]. Sulphated mucins were present only in the mid- to lower pits and MNC, but not in the SMC. Sialylomucins were more clearly demonstrated in all mucin-producing cells with the sensitive method of lectin binding, but were apparent in the SMC and pits and glands after staining with AB pH 2.5 (Fig 5).

Mucins were almost absent from the SMC and the luminal surface By Day 10 p.i. (Fig 5) and there was less intense staining of pit cells, especially the upper regions, consistent with the reduced gene expression of Mu5AC after *H*. *contortus* infection [17]. There was little staining with PAS for total mucins, sulphomucins with HID, both sulphated and sialylated mucins with AB pH 2.5, and with UEA-1 for fucosylated mucins (S7 Fig). The rapidity of these changes was also apparent in sheep receiving transplanted adult worms (Fig 7), in which loss of Muc5AC and expansion of Muc6-producing MNC are clearly seen after 72 hours and in some animals by 24 hours. The reduced mucus layer on the epithelial surface may also be caused by gel-sol transition at pH >4 solubilising the protein [94]. The increased mucosal thickness on Day 10 p.i. and thereafter (Table 1) was largely caused by enlargement of the pits, which became narrow and less clearly defined (Fig 2), although individual cells contained less mucus (Fig 5). These changes persisted until Day 30 p.i. in most animals, but there was evidence of recovery of mucin production in the pit cells and SMC.

The MNC population was expanded to about 50% depth of the glands and stained moderately with PAS by Day 10 p.i. Expansion of the MNC, resulting from their failure to mature from the mucopeptic immature cells to the pepsinogen-producing chief cells, is a well-known effect of abomasal parasitism of sheep [5,10,40] and cattle [95]. As the presence of parietal cells in the progenitor zone, rather than the base of the glands, is necessary for the maintenance and development of the Muc6-secreting MNC [96], inhibition and depletion of parietal cells, as in other gastric pathologies (Menetrière’s disease and pernicious anaemia) [97–98], results in pit enlargement and expansion of the MNC population. This is attributed to stimulation by TGF-α peptides, which promote mucous cell hyperplasia [35,36] and inhibit the differentiation of parietal and zymogenic cells [37].

The role of mucins in parasite expulsion appears to differ in the stomach and intestine. Increased intestinal goblet cell mucin is believed to favour expulsion of some parasite [41,99,100]. This may be the case only in resistant animals [41,101,102], as chronic infection is associated with reduced Muc2, the normal intestinal mucin, and increased secretion of Muc2 with parasite rejection [41,101–103]. In contrast, reduced secretion of Muc5AC in the stomach may be initially permissive for abomasal parasitism, with rapid recovery then favouring parasite expulsion. This is consistent with greater down-regulation of Muc5AC expression in susceptible than resistant sheep, both in a primary infection and subsequent challenge with *H*. *contortus* [104]. Alternatively, loss of the mucus layer may be detrimental to the parasites and favour worm expulsion, as luminal worm stages are sensitive to acid *in vitro* [73,74]. Worms living in the mucus appear to be protected by bicarbonate ions normally secreted by the SMC into the mucus, creating a pH gradient between the neutral epithelial surface and the acidic lumen to protect the mucosa from acid and pepsin damage [105]. The loss of Muc5AC is particularly interesting, as its ectopic expression has been linked to rejection of *Trichuris muris* from the rodent caecum and *Trichinella spiralis* and *Nippostrongylus brasiliensis* from the intestine [106]. It remains to be determined whether the expression of an unusual mucin is of greater significance than the quantity of secreted mucin and its specific properties.

## Conclusions

The parallel changes in host tissues and the numbers of parasites in the abomasal lumen suggest that emerged parasite stages are the key drivers of the pathophysiology and inflammatory response. Restricted local effects around nodules were seen during the tissue phase of larval development. The typical inflammatory response, inhibition of gastric acid secretion, raised serum gastrin and pepsinogen concentrations and loss of parietal cells, enlarged gastric pits containing less mucin and increased numbers of mucous neck cells occurred at the same time after parasite emergence or very rapidly after adult transplantation. Because of the synchrony and speed of effects after adult worm transplantation, as early as 12 hour p.i. in some animals, initiation of the host response could result from chemicals released by parasites in the lumen diffusing across the surface epithelial, aided by components of ES products which increase permeability. Parietal cells appear to be a key target, resulting in secondary increases in serum gastrin, pit elongation, loss of surface mucins and inhibition of chief cell maturation. Inflammation occurs in parallel, and could either itself cause the pathology or exacerbate the direct effects of ES products. Hyperpepsinogenaemia may result from inflammation, as the main accumulation of eosinophils was in the basal area of the mucosa, adjacent to chief cells. The significance of the loss of surface mucins and altered balance of Muc5AC and Muc6 is unclear, as it could either favour or reduce parasite colonisation.

## Supporting information

S1 Fig Gross appearance of a nodule on the gastric pyloric mucosa of sheep killed 5 daysafter infection with 35,000 *Teladorsagia circumcincta* L3. Bar = 0.5 mm.
(DOCX)

S2 Fig **Sections of fundic mucosa showing immunohistochemical staining of parietal cells in tissue from (top) control uninfected lambs and (bottom) from an animal killed 5 days after infection with 35,000 *Teladorsagia circumcincta* L3 (left) and 72 hours after transplantation of 10,000 adult *T*. *circumcincta* (right).** Control tissue showed that parietal cells stained with either anti-TGF-α or anti-pump antibody. Both infected tissues had focal areas with reduced numbers of anti-TGF-α positive parietal cells. (Haematoxylin counterstain). Bar = 200 μm.
(TIF)

S3 Fig Fundic tissues stained with Periodic Acid Schiff for all mucin. Tissues were collected from uninfected sheep and on Days 5, 10, 15, 20 or 30 after infection with 35,000 L3 *Teladorsagia circumcincta*. Bar = 200 μm.
(TIF)

S4 Fig Fundic tissues stained with High Iron Diamine for sulphated mucin. Tissues were collected from uninfected sheep and on Days 5, 10, 15, 20 or 30 after infection with 35,000 L3 *Teladorsagia circumcincta*. Bar = 200 μm.
(TIF)

S5 Fig Fundic tissues stained with Alcian Blue pH 1 for sulphated mucin. Tissues were collected from uninfected sheep and on Days 5, 10, 15, 20 or 30 after infection with 35,000 L3 *Teladorsagia circumcincta*. Bar = 200 μm.
(TIF)

S6 Fig Fundic tissues stained with Alcian Blue pH 2.5 for sialylated and sulphated mucin. Tissues were collected from uninfected sheep and on Days 5, 10, 15, 20 or 30 after infection with 35,000 L3 *Teladorsagia circumcincta*. Bar = 200 μm.
(TIF)

S7 Fig Binding of fluorescently labelled *Ulex europaeus* agglutinin-1 to α-1,2-linked fucose of mucins in the fundus of lambs. Tissues were collected from uninfected sheep and on Days 5, 10, 15, 20 or 30 after infection with 35,000 L3 *Teladorsagia circumcincta*. Bar = 100 μm.
(TIF)

S1 Table Raw data from uninfected sheep and on Days 5, 10, 15, 20 or 30 after infection with 35,000 L3 *Teladorsagia circumcincta*. (DOCX)

S2 Table Raw data from uninfected sheep and 6, 12, 24 or 72 h after transplantation of 10,000 adult *T*. *circumcincta*. (DOCX)
