# Supplementary material for: Abomasal dysfunction and cellular and mucin changes during infection of sheep with larval or adult Teladorsagia circumcincta
Source: PLoS One. 2017 Oct 26;12(10):e0186752. doi: 10.1371/journal.pone.0186752 (PMC5658069; doi:10.1371/journal.pone.0186752)
Supplement: S1 Fig — Bar = 0.5 mm. (DOCX) [file pone.0186752.s001.docx]

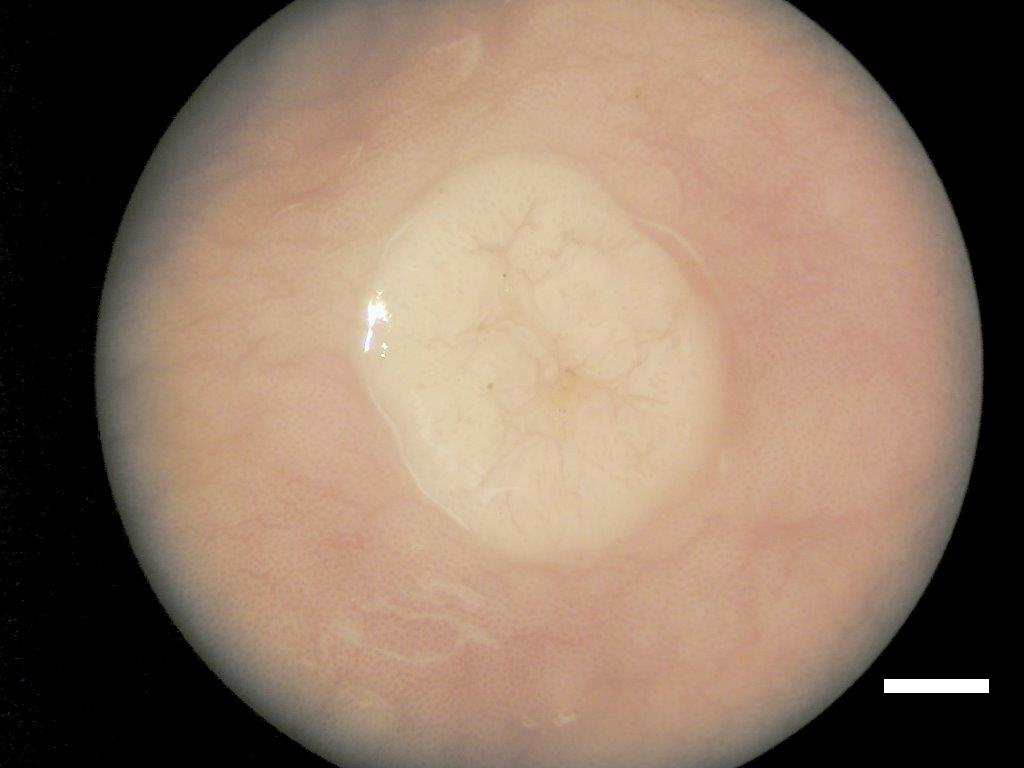


**S1 Fig.** Gross appearance of a nodule on the gastric pyloric mucosa of sheep killed 5 days after infection with 35,000 *Teladorsagia circumcincta* L3. Bar = 1 mm.
