# Supplementary figures and images for: Abomasal dysfunction and cellular and mucin changes during infection of sheep with larval or adult Teladorsagia circumcincta
Source: PLoS One. 2017 Oct 26;12(10):e0186752. doi: 10.1371/journal.pone.0186752 (PMC5658069; doi:10.1371/journal.pone.0186752)

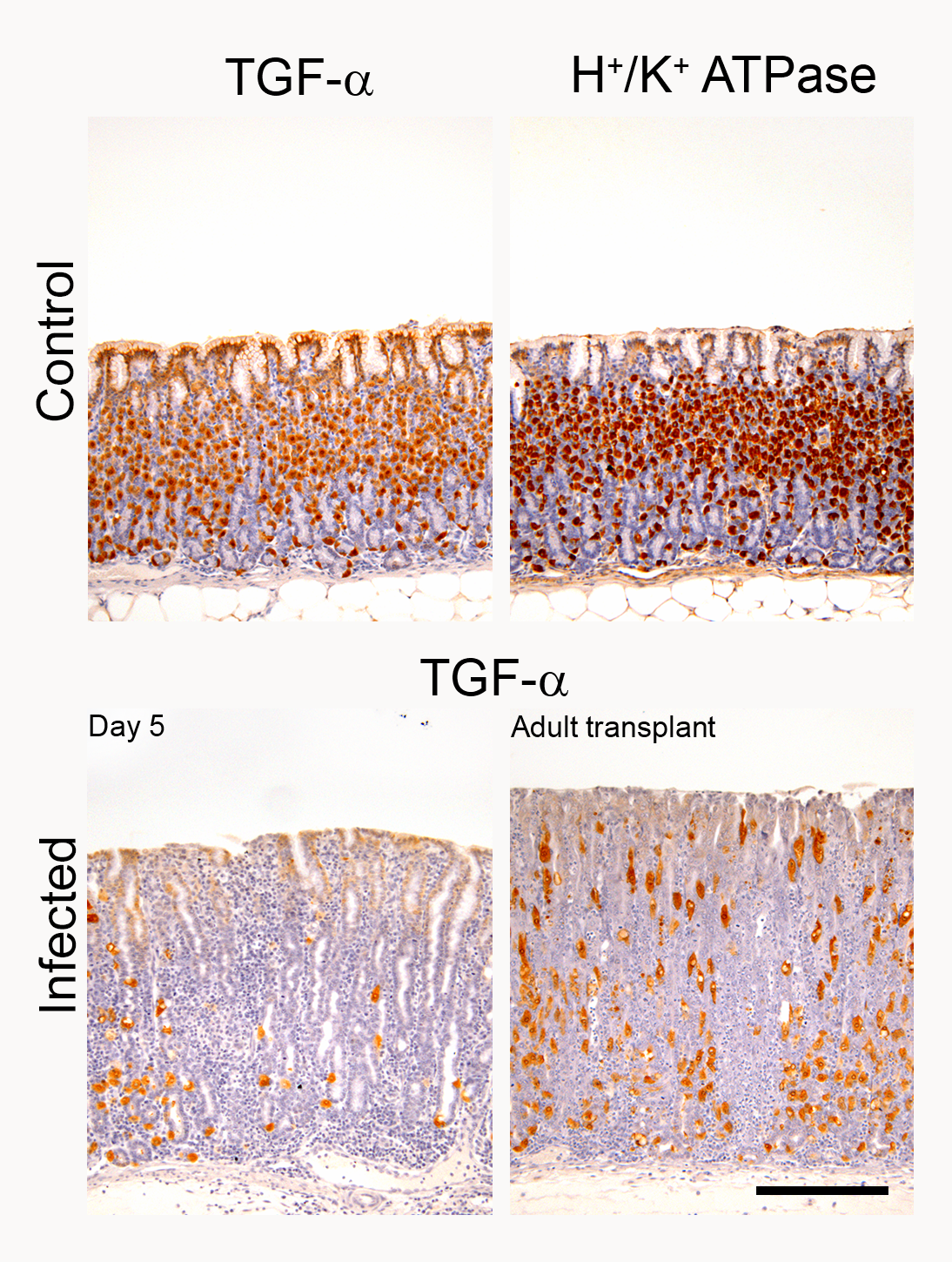

Supplement: S2 Fig — Sections of fundic mucosa showing immunohistochemical staining of parietal cells in tissue from (top) control uninfected lambs and (bottom) from an animal killed 5 days after infection with 35,000 Teladorsagia circumcincta L3 (left) and 72 hours after transplantation of 10,000 adult T. circumcincta (right). Control tissue showed that parietal cells stained with either anti-TGF-α or anti-pump antibody. Both infected tissues had focal areas with reduced numbers of anti-TGF-α positive parietal cells. (Haematoxylin counterstain). Bar = 200 μm. (TIF) [file pone.0186752.s002.tif]

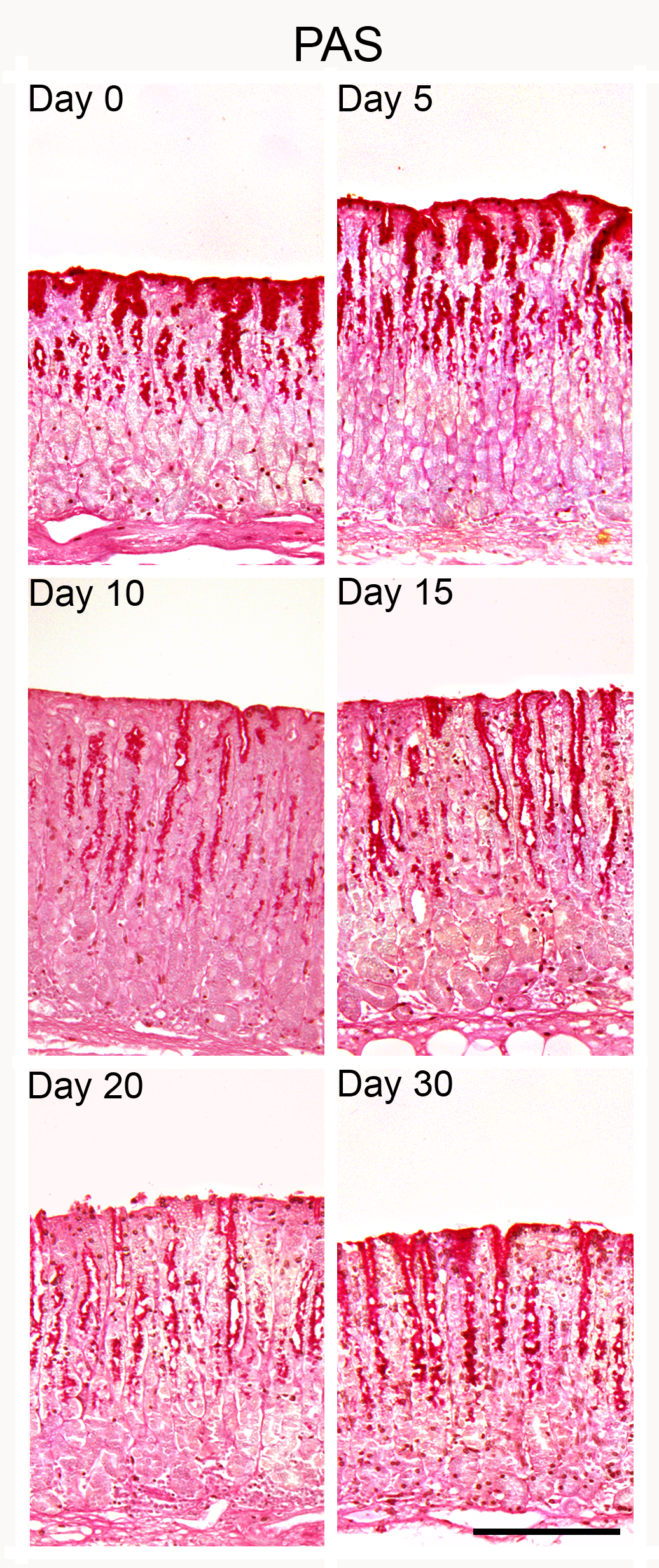

Supplement: S3 Fig — Tissues were collected from uninfected sheep and on Days 5, 10, 15, 20 or 30 after infection with 35,000 L3 Teladorsagia circumcincta. Bar = 200 μm. (TIF) [file pone.0186752.s003.tif]

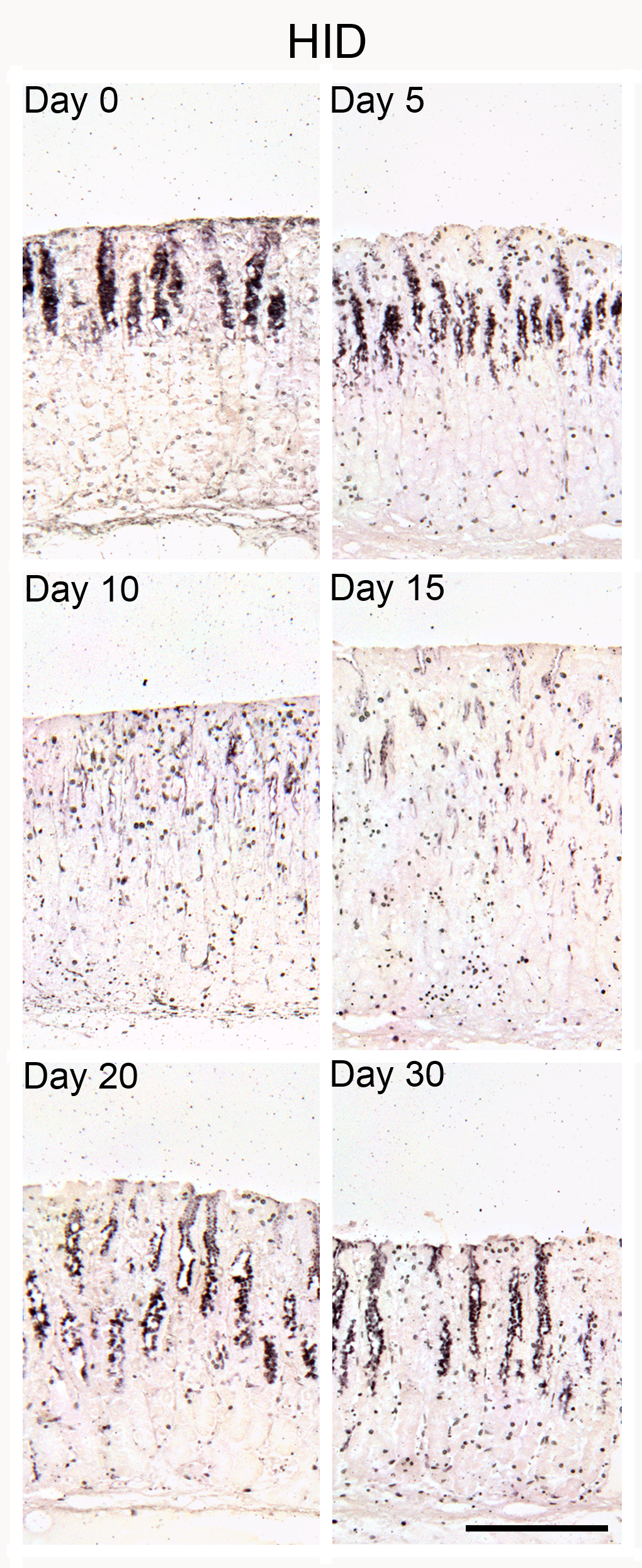

Supplement: S4 Fig — Tissues were collected from uninfected sheep and on Days 5, 10, 15, 20 or 30 after infection with 35,000 L3 Teladorsagia circumcincta. Bar = 200 μm. (TIF) [file pone.0186752.s004.tif]

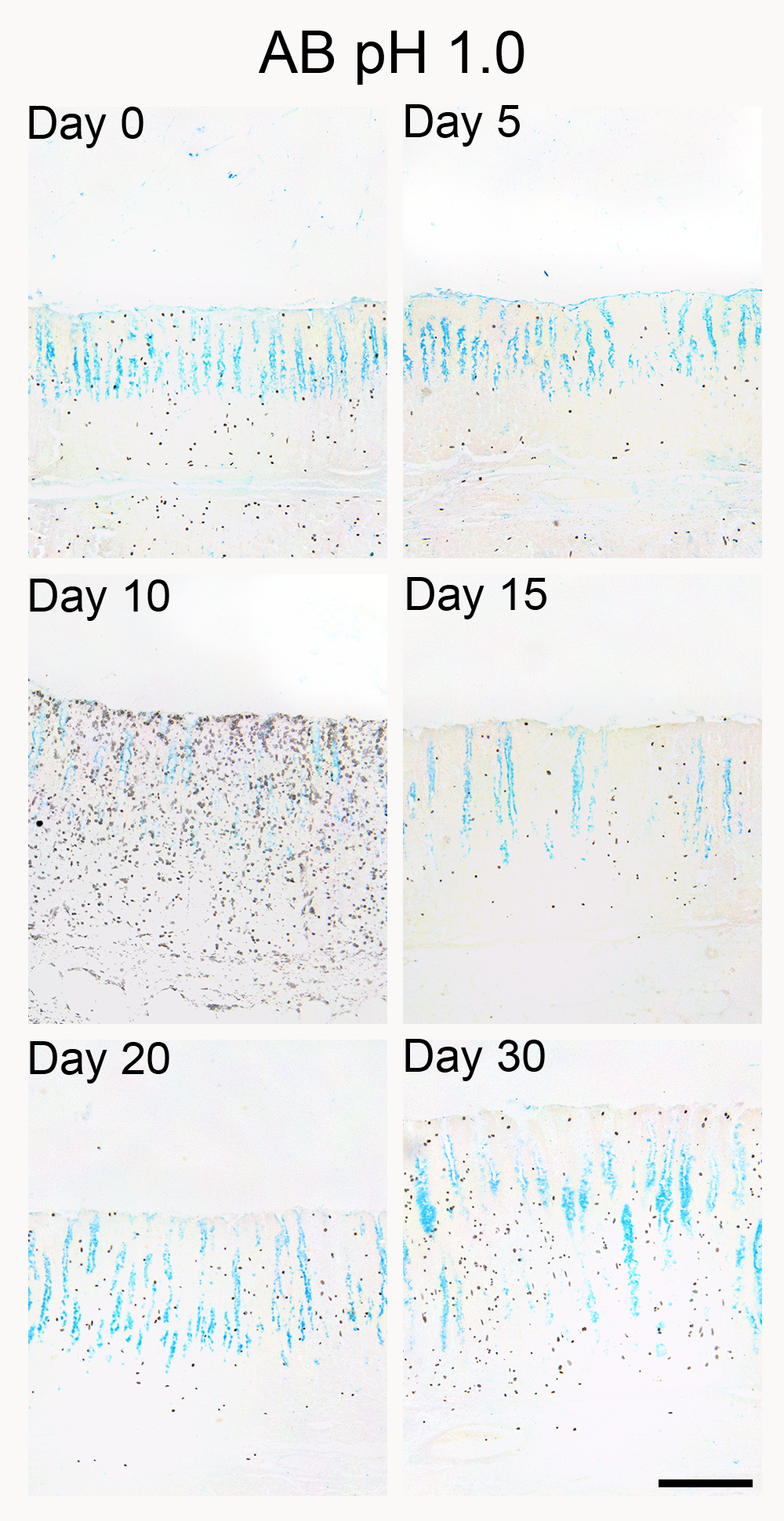

Supplement: S5 Fig — Tissues were collected from uninfected sheep and on Days 5, 10, 15, 20 or 30 after infection with 35,000 L3 Teladorsagia circumcincta. Bar = 200 μm. (TIF) [file pone.0186752.s005.tif]

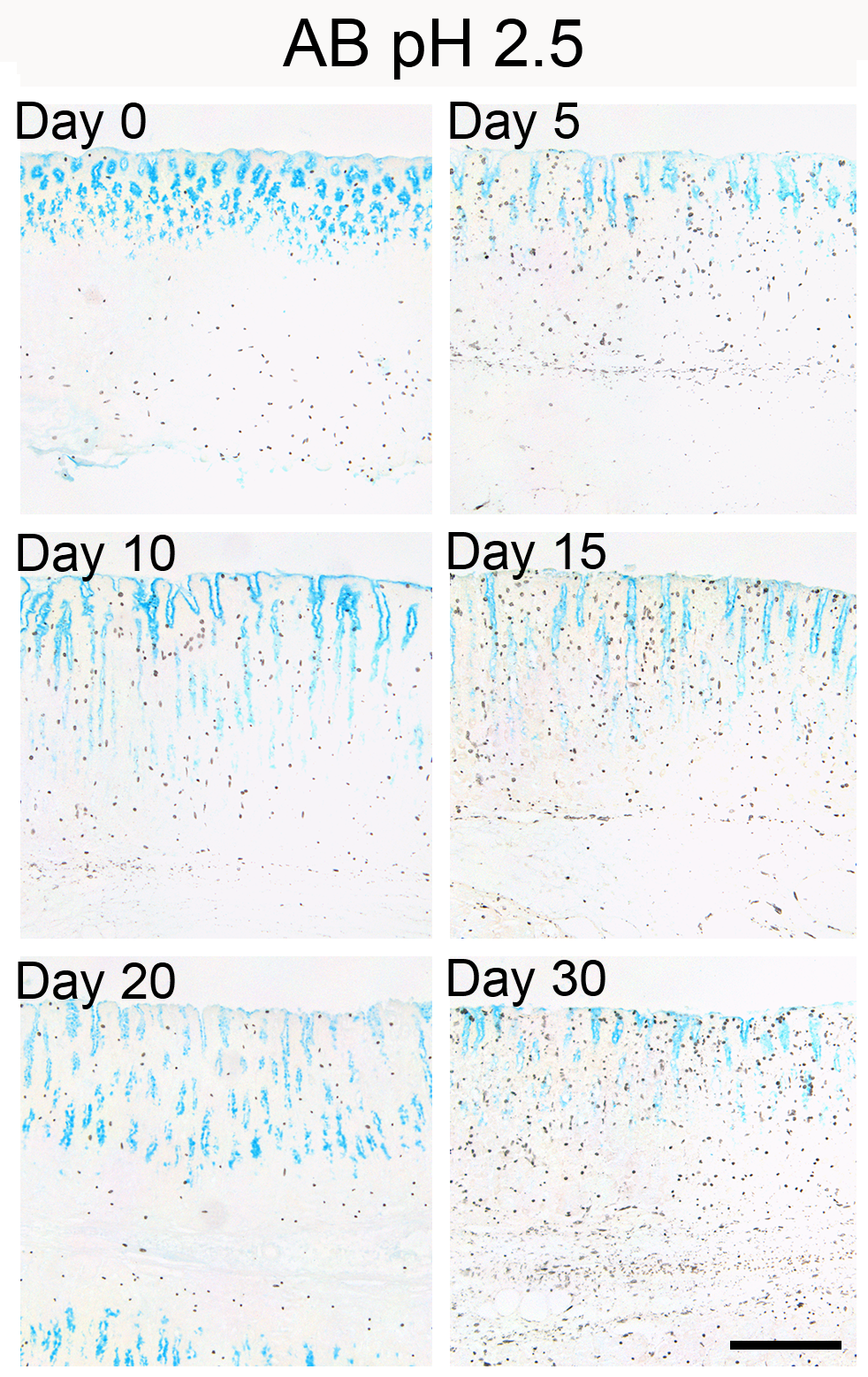

Supplement: S6 Fig — Tissues were collected from uninfected sheep and on Days 5, 10, 15, 20 or 30 after infection with 35,000 L3 Teladorsagia circumcincta. Bar = 200 μm. (TIF) [file pone.0186752.s006.tif]

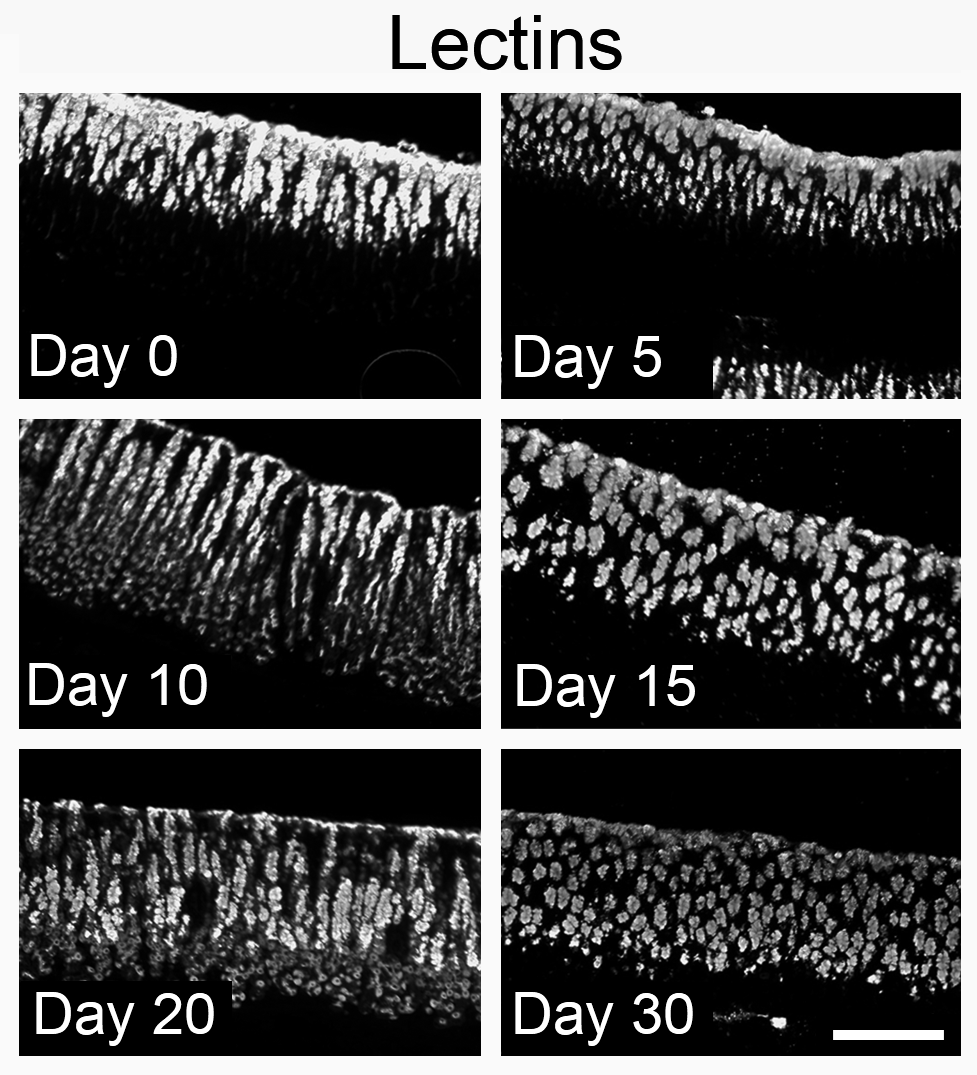

Supplement: S7 Fig — Tissues were collected from uninfected sheep and on Days 5, 10, 15, 20 or 30 after infection with 35,000 L3 Teladorsagia circumcincta. Bar = 100 μm. (TIF) [file pone.0186752.s007.tif]
