# Supplementary material for: Abomasal dysfunction and cellular and mucin changes during infection of sheep with larval or adult Teladorsagia circumcincta
Source: PLoS One. 2017 Oct 26;12(10):e0186752. doi: 10.1371/journal.pone.0186752 (PMC5658069; doi:10.1371/journal.pone.0186752)
Supplement: S1 Table — (DOCX) [file pone.0186752.s008.docx]

**S1 Table. Raw data from uninfected sheep and on Days 5, 10, 15, 20 or 30 after infection with 35,000 L3 *Teladorsagia circumcincta*.**

**S1A. Abomasal pH at necropsy.**

| control | Day 5 | Day 10 | Day 15 | Day 20 | Day 30 |
| --- | --- | --- | --- | --- | --- |
| 2.53 | 2.42 | 6.3 | 4.45 | 3.45 | 3.41 |
| 2.39 | 3.38 | 5.46 | 4.76 | 4.4 | 4.01 |
| 3.52 | 2.66 | 3.33 | 5.1 | 3.22 | 3.09 |
| 3.02 |  | 6.1 | 3.62 | 3.14 | 3.09 |
| 2.22 |  |  |  |  |  |

**S1B. Body weight before infection and at necropsy.** Control sheep marked * killed on Day 5, rest on Day 30.

| control | | Day 5 | | Day 10 | | Day 15 | | Day 20 | | Day 30 | |
| --- | --- | --- | --- | --- | --- | --- | --- | --- | --- | --- | --- |
| Initial | Final | Initial | Final | Initial | Final | Initial | Final | Initial | Final | Initial | Final |
| 25 | 26.8* | 25 | 26.5 | 18 | 19.8 | 24 | 30.3 | 22 | 27.9 | 19 | 27.2 |
| 24 | 27.8* | 16 | 17.9 | 15 | 17.5 | 18 | 23.7 | 24 | 29.2 | 20 | 26.1 |
| 13 | 22.1 | 19 | 19.8 | 17 | 19.3 | 18 | 22.2 | 16 | 20.5 | 22 | 28.4 |
| 14 | 21.5 |  |  | 19 | 23.7 | 23 | 28.1 | 22 | 28.7 | 22 | 28.9 |
| 17 | 25 |  |  |  |  |  |  |  |  |  |  |
| 20 | 32.8 |  |  |  |  |  |  |  |  |  |  |

**S1C. Abomasal weight (g) at necropsy.**

| control | Day 5 | Day 10 | Day 15 | Day 20 | Day 30 |
| --- | --- | --- | --- | --- | --- |
| 122 | 147 | 107 | 172 | 181 | 145 |
| 75 | 82 | 110 | 154 | 142 | 219 |
| 126 | 87 | 146 | 168 | 131 | 145 |
| 86 |  | 126 | 181 | 165 | 143 |
| 132 |  |  |  |  |  |

**S1D. Abomasal mucosal wet weight (g) at necropsy.** A circular punch (11.5 mm diameter) was used to collect tissue. The group (mean ± SEM) and results of ANOVA are also shown.

| control | Day 5 | Day 10 | Day 15 | Day 20 | Day 30 |
| --- | --- | --- | --- | --- | --- |
| 0.1131 | 0.0988 | 0.1604 | 0.1305 | 0.1371 | 0.1384 |
| 0.101 | 0.106 | 0.1625 | 0.1393 | 0.1743 | 0.1879 |
| 0.1001 | 0.140 | 0.1711 | 0.1503 | 0.1283 | 0.14 |
| 0.0944 |  | 0.1703 | 0.1355 | 0.1523 | 0.1429 |
| 0.1115 |  |  |  |  |  |
| 0.1096 |  |  |  |  |  |
| 0.105 ± 0.003 | 0.115 ± 0.013 | 0.166 ± 0.003*** | 0.139 ± 0.004* | 0.148 ± 0.010** | 0.152 ± 0.012*** |

**S1E. Abomasal fundic eosinophil counts per 258 μm wide mucosal tissue column (mean of 5 locations).**

| control | Day 5 | Day 10 | Day 15 | Day 20 | Day 30 |
| --- | --- | --- | --- | --- | --- |
| 0.0 | 0.0 | 36.7 | 25.8 | 5.4 | 12.1 |
| 0.8 | 0.0 | 36.7 | 19.4 | 28.0 | 3.9 |
| 0.2 | 0.0 | 21.4 | 9.67 | 16.9 | 5.9 |
| 0.0 |  | 39.9 | 16.5 | 5.4 | 3.6 |
| 0.0 |  |  |  |  |  |
| 0.04 |  |  |  |  |  |

**S1F. Abomasal pyloric eosinophil counts per 258 μm wide mucosal tissue column (mean of 5 locations).**

| control | Day 5 | Day 10 | Day 15 | Day 20 | Day 30 |
| --- | --- | --- | --- | --- | --- |
| 3.4 | 3.4 | 54.6 | 13.5 | 60.8 | 10.2 |
| 15.4 | 5.6 | 3.5 | 86.8 | 15.3 | 23.3 |
| 13.8 | 15.0 | 46.4 | 16.5 | 43.5 |  |
| 15.4 |  |  | 93.8 | 44.0 |  |
| 62.5 |  |  |  |  |  |

**S1G. Abomasal fundic mast cell counts per 258 μm wide mucosal tissue column (mean of 5 locations).**

| control | Day 5 | Day 10 | Day 15 | Day 20 | Day 30 |
| --- | --- | --- | --- | --- | --- |
| 9.3 | 7.1 | 10.7 | 10.3 | 28 | 29 |
| 8.5 | 7.5 | 14.6 | 7 | 20.2 | 13.5 |
| 9.1 | 8.7 | 6.8 | 22.1 | 6.3 | 21.7 |
| 7.7 |  | 15.73 | 29.2 | 15.1 | 46.7 |
| 6.5 |  |  |  |  |  |
| 6.5 |  |  |  |  |  |

**S1H. Abomasal pyloric mast cell counts per 258 μm wide mucosal tissue column (mean of 5 locations).**

| control | Day 5 | Day 10 | Day 15 | Day 20 | Day 30 |
| --- | --- | --- | --- | --- | --- |
| 20.0 | 11.7 | 14.3 | 15.7 | 32.0 | 17.0 |
| 8.3 | 14.0 | 23.3 | 21.3 | 19.7 | 6.3 |
| 16.3 | 8.0 | 18.3 | 19.3 | 22.3 | 39.0 |
| 16.0 |  | 16.0 | 29.0 | 21.7 | 72.3 |
| 12.7 |  |  |  |  |  |
| 17.0 |  |  |  |  |  |

**S1I. Abomasal fundic parietal cell counts per 258 μm wide mucosal tissue column (mean of 5 locations).**

| control | Day 5 | Day 10 | Day 15 | Day 20 | Day 30 |
| --- | --- | --- | --- | --- | --- |
| 148.8 | 128.2 | 90.9 | 93.7 | 89.2 | 136.1 |
| 140.9 | 160.7 | 121.5 | 94.4 | 121.3 | 71 |
| 143.5 | 172.3 | 107.76 | 75.8 | 140.7 | 163.2 |
| 142.2 |  | 119.4 | 71.4 | 134.5 | 151.2 |
| 150.5 |  |  |  |  |  |
| 122.9 |  |  |  |  |  |

**S1J. Serum gastrin (pM) for individual sheep.**

| Day 0 | Day 1 | Day 2 | Day 3 | Day 4 | Day 5 | Day 6 | Day 7 | Day 8 | Day 9 | Day 10 | Day 11 | Day 12 | Day 13 | Day 14 | Day 15 |
| --- | --- | --- | --- | --- | --- | --- | --- | --- | --- | --- | --- | --- | --- | --- | --- |
| 38 | 51 | 36 | 31 | 32 | 36 | 40 | 63 | 64 | 155 | 139 | 176 | 141 | 207 | 194 | 221 |
| 47 | 38 | 43 | 33 | 44 | 40 | 79 | 112 | 127 | 260 | 284 | 240 | 268 | 336 | 324 | 228 |
| 34 | 59 | 45 | 55 | 74 | 91 |  | 56 | 55 | 68 | 118 | 184 | 224 | 220 | 284 | 328 |
| 38 |  |  |  |  |  | 60 | 135 | 215 | 249 | 157 | 146 | 132 | 140 | 148 | 108 |
| 44 | 35 | 31 | 30 | 39 | 47 | 43 | 80 | 108 | 131 | 179 | 172 | 180 | 181 | 239 | 204 |
| 36 | 41 | 45 | 39 | 53 | 43 | 61 | 96 | 168 | 184 | 228 | 105 | 160 | 124 | 152 | 162 |
| 45 | 39 | 29 | 37 | 29 | 45 | 65 | 78 | 107 | 88 | 104 | 212 | 216 | 142 | 167 | 270 |
| 33 | 75 | 50 | 53 | 73 | 70 | 35 | 38 | 88 | 100 | 196 | 176 | 100 | 104 |  | 208 |
| 42 | 48 | 56 | 48 | 29 | 47 | 37 | 57 | 152 | 184 | 196 | 152 | 132 | 160 | 172 | 128 |
|  | 43 | 35 | 50 | 57 | 73 | 51 | 74 | 70 | 81 | 115 | 188 | 96 | 62 | 100 | 64 |
| 42 | 41 | 50 | 58 | 50 | 47 | 39 | 48 | 112 | 237 | 352 | 116 | 220 | 196 | 248 | 236 |
| 44 | 35 | 33 | 45 | 41 | 39 | 86 | 86 | 120 | 235 | 254 | 192 | 154 | 337 | 285 | 318 |
| 40 | 33 | 37 | 37 | 30 | 50 | 36 | 66 | 104 | 175 | 96 | 176 | 141 | 207 | 194 | 221 |
| 62 | 27 | 32 | 26 | 38 | 74 | 50 | 71 | 113 | 208 | 220 | 240 | 268 | 336 | 324 | 228 |
| 41 | 43 | 29 | 39 | 32 | 32 | 24 | 43 | 157 | 201 | 198 |  |  |  |  |  |
| 29 | 75 | 68 | 66 | 58 | 74 | 88 | 136 | 100 | 158 | 108 |  |  |  |  |  |
| 54 | 23 | 25 | 24 | 26 | 28 |  |  |  |  |  |  |  |  |  |  |
| 35 | 32 | 60 | 25 | 46 | 24 |  |  |  |  |  |  |  |  |  |  |
| 36 | 21 | 19 | 33 | 26 | 29 |  |  |  |  |  |  |  |  |  |  |
| 42 | 22 | 28 | 28 | 28 | 72 |  |  |  |  |  |  |  |  |  |  |
| 28 |  |  |  |  |  |  |  |  |  |  |  |  |  |  |  |
| 59 |  |  |  |  |  |  |  |  |  |  |  |  |  |  |  |
| 18 |  |  |  |  |  |  |  |  |  |  |  |  |  |  |  |
| 26 |  |  |  |  |  |  |  |  |  |  |  |  |  |  |  |
| 15 |  |  |  |  |  |  |  |  |  |  |  |  |  |  |  |
| 27 |  |  |  |  |  |  |  |  |  |  |  |  |  |  |  |

| Day 17 | Day 19 | Day 20 | Day 21 | Day 23 | Day 25 | Day 27 | Day 30 |
| --- | --- | --- | --- | --- | --- | --- | --- |
| 96 | 52 | 46 | 60 | 44 | 40 | 44 | 64 |
| 112 | 260 | 174 |  |  |  |  |  |
| 165 | 108 | 157 | 68 | 52 | 60 | 59 | 80 |
| 227 | 124 | 215 | 52 | 88 | 88 | 96 | 72 |
| 68 |  |  |  |  |  |  |  |
| 116 |  |  |  |  |  |  |  |
| 216 |  |  |  |  |  |  |  |
| 109 |  |  |  |  |  |  |  |

**S1K. Abomasal fundic mucosal thickness for PAS and HID stained sections (duplicates per slide).**

| # | control | | Day 5 | | Day 10 | | Day 15 | | Day 20 | | Day 30 | |
| --- | --- | --- | --- | --- | --- | --- | --- | --- | --- | --- | --- | --- |
|  | PAS | HID | PAS | HID | PAS | HID | PAS | HID | PAS | HID | PAS | HID |
| 1 | 397, 410 | 495, 536 | 346, 358 | 350, 341 | 547, 564 | 632, 625 | 582, 576 | 600, 584 | 517, 504 | 526, 504 | 496, 474 | 510, 502 |
| 2 | 383, 385 | 439, 407 | 604, 609 | 524, 509 | 639, 691 | 701, 698 | 526, 523 | 456, 454 | 571, 533 | 570, 552 | 504, 502 | 511, 522 |
| 3 | 376, 385 | 442, 457 | 348, 321 | 334, 341 | 625, 627 | 591, 611 | 630, 635 | 620, 597 | 559, 561 |  | 533, 524 | 504, 504 |
| 4 | 456, 443 | 419, 428 |  |  | 578, 571 | 584, 587 | 547, 542 | 613, 630 | 511, 516 | 538, 534 | 658, 685 | 837, 893 |
| 5 | 476, 467 | 452, 430 |  |  |  |  |  |  |  |  |  |  |
| 6 | 440, 435 | 477, 427 |  |  |  |  |  |  |  |  |  |  |

**S1L. Serum pepsinogen (pM) for individual sheep before and after infection.**

| Day 0 | Day 1 | Day 2 | Day 3 | Day 4 | Day 5 | Day 6 | Day 7 | Day 8 | Day 9 | Day 10 | Day 11 | Day 12 | Day 13 | Day 14 | Day 15 |
| --- | --- | --- | --- | --- | --- | --- | --- | --- | --- | --- | --- | --- | --- | --- | --- |
| 0 | 0.49 | 1.25 | 1.49 | 2.02 | 1.90 | 1.87 | 0.85 | 2.17 | 3.85 | 4.41 | 4.22 | 3.04 | 1.63 | 3.17 | 3.19 |
| 0 | 1.50 | 0.00 | 1.56 | 1.80 | 3.26 | 1.55 | 1.66 | 2.01 | 2.94 | 2.89 | 2.83 | 4.31 | 2.96 | 3.12 | 5.30 |
| 1.47 | 0.49 | 2.58 | 1.19 | 1.13 | 0.19 | 1.48 | 3.02 | 1.57 | 2.55 | 3.35 | 3.14 | 6.45 | 5.64 | 5.64 | 11.41 |
| 0.95 |  |  |  |  |  | 0.94 | 2.35 | 3.23 | 5.13 | 5.23 | 7.51 | 8.19 | 8.19 | 6.48 | 6.56 |
| 1.27 | 0.76 | 1.19 | 0.57 | 2.04 | 0.27 | 1.35 | 0.59 | 1.22 | 1.47 | 1.98 | 1.48 | 1.69 | 4.93 | 4.81 | 4.87 |
| 1.52 | 1.46 | 0.09 | 0.48 | 1.36 | 0.00 | 1.57 | 1.56 | 2.91 | 4.88 | 3.64 | 7.65 | 5.66 | 7.22 | 6.80 | 0.11 |
| 0.10 | 1.24 | 0.72 | 1.91 | 1.31 | 1.81 | 2.60 | 2.09 | 1.86 | 3.39 | 0.26 | 7.30 | 8.31 | 5.58 | 7.23 | 2.72 |
| 0.40 | 0.72 | 2.68 | 1.96 | 1.68 | 0.79 | 2.15 | 1.59 | 0.36 | 2.50 | 4.80 | 5.29 | 4.44 | 2.51 | 1.86 | 1.90 |
| 0.78 | 0.84 | 3.44 | 0.19 | 0.64 | 0.79 | 0.97 | 0.73 | 2.79 | 3.56 | 5.31 | 3.87 | 1.86 | 1.38 | 2.86 | 1.73 |
|  | 2.28 | 0.09 | 1.94 | 1.28 | 2.46 | 0.39 | 1.68 | 0.55 | 1.46 | 1.88 | 1.05 | 1.16 | 2.14 | 1.22 | 1.75 |
| 0.77 | 0.37 | 0.77 | 0.21 | 1.45 | 1.67 | 0.47 | 0.25 | 1.14 | 1.23 | 1.47 | 1.67 | 0.21 |  | 0.20 | 0.86 |
| 0.80 | 0.77 | 1.34 | 1.499 | 1.14 | 0.84 | 0.62 | 0.45 | 1.37 | 1.92 | 1.46 | 1.05 | 0.85 | 1.34 | 0.00 | 1.64 |
| 1.10 | 0.91 | 0.19 | 0.96 | 1.07 | 0.00 | 0.97 | 0.63 | 0.00 | 0.90 | 1.46 |  |  |  |  |  |
| 0.47 | 1.21 | 1.45 | 0.92 | 0.65 | 0.64 | 3.45 | 1.21 | 2.67 | 1.25 | 2.12 |  |  |  |  |  |
| 0.56 | 0.74 | 1.01 | 0.00 | 0.85 | 0.83 |  | 3.61 | 1.58 | 2.60 | 2.06 |  |  |  |  |  |
| 1.44 | 0.48 | 0.73 | 0.95 | 0.47 | 0.64 | 1.09 | 1.28 | 2.57 | 3.93 | 3.85 |  |  |  |  |  |
| 2.38 | 0.80 | 0.00 | 1.15 | 0.79 | 0.67 |  |  |  |  |  |  |  |  |  |  |
| 0.00 | 1.52 | 1.89 | 3.81 | 1.03 | 0.36 |  |  |  |  |  |  |  |  |  |  |
| 3.27 | 3.35 | 0.90 | 0.84 | 1.08 | 0.68 |  |  |  |  |  |  |  |  |  |  |
| 1.04 |  |  |  |  |  |  |  |  |  |  |  |  |  |  |  |
| 0 |  |  |  |  |  |  |  |  |  |  |  |  |  |  |  |
| 1.47 |  |  |  |  |  |  |  |  |  |  |  |  |  |  |  |
| 0.72 |  |  |  |  |  |  |  |  |  |  |  |  |  |  |  |
| 0.44 |  |  |  |  |  |  |  |  |  |  |  |  |  |  |  |
| 2.61 |  |  |  |  |  |  |  |  |  |  |  |  |  |  |  |
| 1.79 |  |  |  |  |  |  |  |  |  |  |  |  |  |  |  |

| Day 17 | Day 19 | Day 20 | Day 21 | Day 23 | Day 25 | Day 27 | Day 30 |
| --- | --- | --- | --- | --- | --- | --- | --- |
| 4.32 | 2.44 | 6.93 | 2.22 | 1.78 | 1.42 | 1.60 | 2.10 |
| 5.94 | 1.36 | 3.35 | 1.56 | 1.93 | 2.21 | 1.21 | 1.62 |
| 3.33 | 1.19 | 4.70 | 1.83 | 2.13 | 2.21 | 2.06 | 2.392 |
| 3.10 | 0.83 | 2.11 |  | 1.38 | 0.84 | 1.41 | 1.80 |
| 1.22 |  |  |  |  |  |  |  |
| 2.01 |  |  |  |  |  |  |  |
| 1.42 |  |  |  |  |  |  |  |
| 0 |  |  |  |  |  |  |  |
