# Supplementary material for: Abomasal dysfunction and cellular and mucin changes during infection of sheep with larval or adult Teladorsagia circumcincta
Source: PLoS One. 2017 Oct 26;12(10):e0186752. doi: 10.1371/journal.pone.0186752 (PMC5658069; doi:10.1371/journal.pone.0186752)
Supplement: S2 Table — (DOCX) [file pone.0186752.s009.docx]

**S2 Table. Raw data from uninfected sheep and after transplantation of 10,000 adult *T. circumcincta*.**

**S2A. Body weights 2-3 weeks before adult worm transplant and at necropsy.**

| control | | 6 h | | 12 h | | 24 h | | 72 h | |
| --- | --- | --- | --- | --- | --- | --- | --- | --- | --- |
| Initial | Final | Initial | Final | Initial | Final | Initial | Final | Initial | Final |
| 28 | 33.3 | 26 | 33 | 29 | 35 | 24 | 29 | 23 | 23 |
| 30 | 36.9 | 24 | 29 | 29 | 33 | 25 | 28 | 25 | 29 |
| 30 | 35 | 27 | 31 | 27 | 31 | 27 | 29.2 | 25 | 28.6 |

**S2B. Weight of abomasum (g) at necropsy.**

| control | 6 h | 12 h | 24 h | 72 h |
| --- | --- | --- | --- | --- |
| 128 | 156 | 132 |  | 119 |
| 145 | 151 | 128 | 127 | 151 |
| 138 | 141 | 133 | 140 | 119 |

**S2C. Abomasal mucosal wet weight (g) at necropsy.** A circular punch (11.5 mm diameter) was used to collect tissue.

| control | 6 h | 12 h | 24 h | 72 h |
| --- | --- | --- | --- | --- |
| 0.1335 | 0.1243 | 0.2036 | 0.1633 | 0.1514 |
| 0.1252 | 0.1671 | 0.1815 | 0.1976 | 0.1847 |
| 0.1125 | 0.1344 | 0.1335 | 0.1233 | 0.1967 |

**S2D. Abomasal fundic parietal cell counts per 258 μm wide mucosal tissue column (mean of 5 locations).**

| control | 6 h | 12 h | 24 h | 72 h |
| --- | --- | --- | --- | --- |
| 153.2 | 171.8 | 148.6 | 154.1 | 113.3 |
| 165 | 167.2 | 167.3 | 192.7 | 149.3 |
| 163.3 | 151.4 | 148.2 | 176.4 | 194.4 |

**S2E. Abomasal fundic eosinophil counts per 258 μm wide mucosal tissue column (mean of 5 locations).**

| control | 6 h | 12 h | 24 h | 72 h |
| --- | --- | --- | --- | --- |
| 0.16 | 0.98 | 1.95 | 4.73 | 33.8 |
| 0.2 | 14.9 | 13.05 | 3.94 | 15.25 |
| 0.16 | 1.06 | 1.46 | 0.56 | 1.75 |

**S2F. Abomasal fundic mast cell counts per 258 μm wide mucosal tissue column (mean of 5 locations).**

| control | 6 h | 12 h | 24 h | 72 h |
| --- | --- | --- | --- | --- |
| 5.220 | 2.10 | 3.12 | 4.50 | 2.00 |
| 4.390 | 3.96 | 2.75 | 8.30 | 2.54 |
| 2.220 | 2.09 | 3.80 | 5.60 | 3.60 |

**S2G. Abomasal fundic mucosal thickness for PAS and HID stained sections (duplicates per slide).**

| # | control | | 6 h | | 12 h | | 24 h | | 72 h | |
| --- | --- | --- | --- | --- | --- | --- | --- | --- | --- | --- |
|  | PAS | HID | PAS | HID | PAS | HID | PAS | HID | PAS | HID |
| 1 | 563, 555 | 612, 616 | 638, 622 | 812, 830 | 601, 610 | 583, 566 | 685, 696 | 657, 614 | 594, 567 | 767, 720 |
| 2 | 528, 535 | 508, 506 | 550, 557 | 511, 539 | 881, 896 | 685, 686 | 638, 661 | 726, 711 | 807, 796 | 744, 730 |
| 3 | 468, 489 | 536, 569 | 481, 491 | 503, 502 | 491, 441 | 455, 463 | 395, 405 | 544, 550 | 817, 776 | 682, 647 |

**S2H. Serum pepsinogen uninfected controls (iU) (3 sheep).**

| Time (h) | 1 | 2 | 3 |
| --- | --- | --- | --- |
| 0 | 0.000 | 0.930 | 0.930 |
| 6 | 1.110 | 1.230 | 0.990 |
| 9 | 1.360 | 1.600 | 0.730 |
| 12 | 1.480 | 0.990 | 0.490 |
| 15 | 1.110 | 0.620 | 1.030 |
| 21 | 0.690 | 0.920 | 0.800 |
| 24 | 1.610 | 0.570 | 0.340 |
| 30 | 1.112 | 1.191 | 1.773 |
| 37 | 1.366 | 0.831 | 1.201 |
| 45 | 1.363 | 1.569 | 1.270 |
| 48 | 1.034 | 0.388 | 1.329 |
| 54 | 1.718 | 1.163 | 0.936 |
| 60 | 0.811 | 1.028 | 1.082 |
| 72 | 1.100 | 1.389 | 0.577 |

**S2I. Serum pepsinogen infected sheep (12 sheep).**

| Time | 1 | 2 | 3 | 4 | 5 | 6 | 7 | 8 | 9 | 10 | 11 | 12 |
| --- | --- | --- | --- | --- | --- | --- | --- | --- | --- | --- | --- | --- |
| 0 | 0.950 | 0.720 | 0.450 | 1.340 | 0.540 | 0.0 | 0.450 | 0.990 | 1.080 | 0.90 | 1.080 | 0.630 |
| 3 | 0.000 | 1.060 | 0.520 | 1.210 | 0.950 | 0.43 | 0.860 | 0.950 | 0.780 | 1.03 | 1.380 | 0.170 |
| 6 | 0.890 | 0.830 | 0.890 | 1.220 | 1.240 | 0.73 | 0.980 | 1.320 | 0.490 | 0.98 | 0.570 | 1.300 |
| 9 | 1.610 | 1.070 | 1.320 | 1.320 | 2.640 | 0.66 | 1.160 | 1.650 | 1.650 |  |  |  |
| 12 | 2.150 | 1.610 | 1.260 | 2.510 |  | 1.08 | 0.900 | 2.960 | 3.050 |  |  |  |
| 15 | 1.830 | 0.780 | 0.430 | 1.910 | 4.170 | 2.09 |  |  |  |  |  |  |
| 18 | 1.830 | 2.090 | 0.960 | 2.610 | 3.480 | 0.78 |  |  |  |  |  |  |
| 21 | 2.350 | 1.390 | 0.870 | 3.220 | 3.560 | 1.22 |  |  |  |  |  |  |
| 24 | 1.480 | 1.560 | 2.110 | 3.170 | 2.780 | 1.22 |  |  |  |  |  |  |
| 30 | 1.630 | 1.630 | 0.980 |  |  |  |  |  |  |  |  |  |
| 36 | 1.950 | 1.300 | 0.810 |  |  |  |  |  |  |  |  |  |
| 42 | 2.110 | 1.710 | 1.380 |  |  |  |  |  |  |  |  |  |
| 48 | 2.280 | 1.630 | 1.300 |  |  |  |  |  |  |  |  |  |
| 62 | 1.950 | 2.030 | 1.140 |  |  |  |  |  |  |  |  |  |
| 72 | 2.190 | 2.440 | 0.650 |  |  |  |  |  |  |  |  |  |

**S2J. Abomasal pH uninfected controls (3 sheep).**

| Time (h) | 1 | 2 | 3 |
| --- | --- | --- | --- |
| 0 | 2.74 | 2.93 | 2.48 |
| 3 | 2.300 | 2.810 | 2.710 |
| 6 | 2.14 | 2.65 | 2.36 |
| 9 | 2.53 | 2.62 | 2.69 |
| 12 | 2.49 | 2.60 | 2.49 |
| 15 | 2.45 | 2.82 | 2.99 |
| 21 | 2.70 | 3.11 | 3.16 |
| 24 | 2.78 | 2.69 | 2.69 |
| 30 | 2.81 | 2.65 | 3.01 |
| 37 |  | 2.52 | 2.44 |
| 45 | 2.94 | 2.57 | 2.61 |
| 48 | 2.91 | 2.50 | 2.75 |
| 54 | 2.81 | 2.79 | 2.79 |
| 60 | 2.65 | 2.45 | 2.29 |
| 72 | 2.71 | 2.59 | 2.98 |

**S2K. Abomasal pH infected sheep (12 sheep).**

| Time | 1 | 2 | 3 | 4 | 5 | 6 | 7 | 8 | 9 | 10 | 11 | 12 |
| --- | --- | --- | --- | --- | --- | --- | --- | --- | --- | --- | --- | --- |
| 0 |  |  |  |  | 2.830 | 2.770 | 2.730 | 3.070 | 2.730 | 2.400 | 2.730 | 3.030 |
| 6 | 2.270 | 2.900 | 2.080 | 2.520 | 2.200 | 2.470 | 2.800 | 3.250 | 3.000 | 3.700 | 2.890 | 2.340 |
| 9 | 2.570 | 3.770 |  |  |  | 3.180 | 2.910 | 3.280 |  |  |  |  |
| 12 | 2.270 | 3.270 | 1.950 | 3.590 |  | 3.100 | 2.230 | 2.790 | 3.060 |  |  |  |
| 15 | 2.420 | 2.720 | 2.170 | 3.310 |  | 3.320 |  |  |  |  |  |  |
| 18 | 2.610 | 2.470 | 2.280 | 3.160 |  |  |  |  |  |  |  |  |
| 21 | 2.700 | 3.850 | 2.990 | 3.060 | 3.950 | 3.430 |  |  |  |  |  |  |
| 24 | 2.880 | 2.930 | 1.910 | 3.590 | 4.120 | 2.390 |  |  |  |  |  |  |
| 30 | 3.190 | 3.160 | 2.410 |  |  |  |  |  |  |  |  |  |
| 36 | 2.860 | 3.500 | 2.360 |  |  |  |  |  |  |  |  |  |
| 42 | 3.120 | 4.020 |  |  |  |  |  |  |  |  |  |  |
| 46 | 3.090 | 3.750 | 2.810 |  |  |  |  |  |  |  |  |  |
| 49 | 3.030 | 3.770 | 2.660 |  |  |  |  |  |  |  |  |  |
| 53 | 3.100 | 4.080 | 2.630 |  |  |  |  |  |  |  |  |  |
| 62 | 3.010 | 4.030 | 2.790 |  |  |  |  |  |  |  |  |  |
| 72 | 4.020 | 5.400 | 2.990 |  |  |  |  |  |  |  |  |  |
